# LncRNA ENSSSCG00000035331 Alleviates Hippocampal Neuronal Ferroptosis and Brain Injury Following Porcine Cardiopulmonary Resuscitation by Regulating the miR‐let7a/GPX4 Axis

**DOI:** 10.1111/cns.70377

**Published:** 2025-04-16

**Authors:** Mao Zhang, Wenbin Zhang, Ziwei Chen, Lu He, Qijiang Chen, Pin Lan, Lulu Li, Xianlong Wu, Xingui Wu, Jiefeng Xu

**Affiliations:** ^1^ Department of Emergency Medicine, Second Affiliated Hospital Zhejiang University School of Medicine Hangzhou China; ^2^ Zhejiang Key Laboratory of Trauma, Burn, and Medical Rescue Hangzhou China; ^3^ Zhejiang Province Clinical Research Center for Emergency and Critical Care Medicine Hangzhou China; ^4^ Department of Intensive Care Medicine The First Hospital of Ninghai Ningbo China; ^5^ Department of Emergency Medicine Fifth Affiliated Hospital of Wenzhou Medical University, Lishui Central Hospital Lishui China; ^6^ Department of Emergency Medicine, First Affiliated Hospital Zhejiang University School of Medicine Hangzhou China; ^7^ Department of Emergency Medicine Taizhou First People's Hospital Taizhou China; ^8^ Institute of Pediatrics, Guangdong Provincial Key Laboratory of Research in Structural Birth Defect Disease Guangzhou China; ^9^ Guangzhou Women and Children's Medical Center Guangzhou Medical University Guangzhou China

**Keywords:** brain injury, cardiac arrest, ENSSSCG00000035331, hippocampal neuronal ferroptosis, miR‐let7a, sulforaphane

## Abstract

**Background:**

Following successful cardiopulmonary resuscitation, those survivors of cardiac arrest (CA) often suffer from severe brain injury, and the latter can result in significant mortality and morbidity. Emerging evidence implicates that ferroptosis is involved in the pathogenesis of post‐resuscitation brain injury, and its regulatory mechanisms remain to be investigated. Recently, some studies manifested that long noncoding RNAs could be critical regulators of cell ferroptosis in diverse ischemia–reperfusion injuries of vital organs. This study was designed to explore the role and mechanism of a newly screened long noncoding RNA ENSSSCG00000035331 in alleviating post‐resuscitation hippocampal neuronal ferroptosis and further investigate its potential regulation by a novel antioxidant sulforaphane.

**Methods and Results:**

Healthy male pigs and mice were used to establish the models of CA and resuscitation in vivo. A hypoxia/reoxygenation (H/R) model using primary porcine hippocampal neurons was constructed to replicate post‐resuscitation brain injury in vitro. We found that the expression of ENSSSCG00000035331 was significantly decreased in the post‐resuscitation impaired hippocampus using RNA sequencing analysis and verification. Subsequently, ENSSSCG00000035331 overexpression significantly reduced ferroptosis‐related ferrous iron and reactive oxygen species production while markedly increased glutathione and further alleviated post‐resuscitation brain injury. Mechanistically, ENSSSCG00000035331 interacted with miR‐let7a, then inhibited its binding with glutathione peroxidase 4 (GPX4) mRNA and finally promoted the recovery of the latter's translation after H/R stimulation. In addition, sulforaphane treatment significantly increased ENSSSCG00000035331 and GPX4 expression while markedly decreased miR‐let7a expression and hippocampal neuronal ferroptosis and finally alleviated post‐resuscitation brain injury.

**Conclusions:**

Our findings highlighted that ENSSSCG00000035331 was a critical regulator of hippocampal neuronal ferroptosis after CA and resuscitation by targeting the miR‐let7a/GPX4 axis, and additionally, sulforaphane might be a promising therapeutic agent for alleviating post‐resuscitation brain injury by regulating the signaling axis mentioned above.

## Graphical abstract

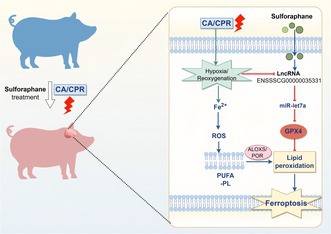

## 1 Introduction

Cardiac arrest (CA) survivors frequently suffer from severe brain injury after restoring spontaneous circulation from cardiopulmonary resuscitation (CPR) [1]. Post‐resuscitation brain injury has become the primary cause of mortality and morbidity at the stage of post‐resuscitation care in CA victims [2]. Although therapeutic hypothermia is always recommended to alleviate post‐resuscitation brain injury in clinical guidelines; however, its therapeutic efficacy is limited [3, 4]. Currently, the pathogenesis of post‐resuscitation brain injury is still required to explore and further develop its effective therapeutic strategies.

Ferroptosis, recently identified as a novel form of regulatory cell death, is characterized by an iron‐dependent and caspase‐independent mode of cell death outside of apoptosis and acts as a central regulator in ischemia–reperfusion injury (IRI) across various organs [5, 6]. Following IRI, ferrous iron (Fe^2+^) is abnormally elevated in those injured cells and then promotes the production of reactive oxygen species (ROS) via Fenton reactions, and the latter facilitates lipid peroxidation of polyunsaturated fatty acids in those biomembranes to produce lipid peroxides and finally leads to the occurrence of cell ferroptosis [7]. Additionally, glutathione peroxidase 4 (GPX4) is acknowledged as a key molecule that negatively regulates the process of ferroptosis by converting lipid peroxides into nontoxic lipid alcohols via the oxidation of glutathione (GSH) to glutathione disulfide [8]. Recent studies have highlighted the prominent role of ferroptosis in brain IRI, particularly suggesting its involvement in the pathogenesis of post‐resuscitation brain injury [9, 10]. However, the regulatory mechanisms of ferroptosis and its potential intervention strategies in post‐resuscitation brain injury require further investigations.

Recent studies have consistently demonstrated that the long noncoding RNAs (lncRNAs) which act as competing endogenous RNAs are integral to numerous biological processes by interacting with microRNAs [11, 12]. This competing mechanism is vital in pathological conditions including diverse IRI events, and highlights the important role of lncRNAs in regulating cellular homeostasis and stress response. Especially, some studies have confirmed that lncRNAs could regulate the process of cell ferroptosis and further affect the pathological progression of regional IRI of multiple vital organs, such as the brain, heart, liver, and kidney [13, 14, 15, 16]. Nonetheless, the exploration of key lncRNAs that regulate brain ferroptosis after CA and resuscitation remains to be investigated.

In this study, we focused on the hippocampus, a representative vulnerable region of the brain following CA and resuscitation. Then, we employed whole transcriptome sequencing to successfully identify the top differentially expressed lncRNA ENSSSCG00000035331 in the post‐resuscitation impaired hippocampus in a clinically relevant, large‐animal model. We proceeded to investigate its role in the process of hippocampal neuronal ferroptosis after hypoxia/reoxygenation (H/R) in vitro and CA and resuscitation in vivo. Consequently, we demonstrated that ENSSSCG00000035331 overexpression significantly inhibited hippocampal neuronal ferroptosis by recovering the translation of GPX4 mRNA via interacting with miR‐let7a. Furthermore, we confirmed that a specific antioxidant, sulforaphane (SFN) significantly alleviated post‐resuscitation brain injury possibly by inhibiting ferroptosis via regulating the ENSSSCG00000035331/miR‐let7a/GPX4 axis.

## 2 Methods

### 2.1 Ethical Statement

All experimental animals were managed in strict accordance with the “Principles of Laboratory Animal Care” established by the National Medical Research Association and the “Guidelines for the Care and Use of Laboratory Animals” issued by the Institute of Laboratory Animal Resources, thereby ensuring humane treatment throughout the experiment. This experimental protocol received formal approval from the Institutional Animal Care and Use Committee of the Second Affiliated Hospital, Zhejiang University School of Medicine (2024034).

### 2.2 Sex as a Biological Variable

In this study, healthy male domestic pigs (4–6 m, 35–42 kg) were purchased from Shanghai Jiagan Biotechnology Inc. (Shanghai, China), and healthy male C57BL/6 mice (8–12 w, 20–25 g) were purchased from Zhejiang Vital River Laboratory Animal Technology Co. Ltd. (Zhejiang, China). Previous research has shown that female animals exhibit better preservation of hemodynamic parameters and less myocardial damage following hemorrhage and resuscitation from circulatory arrest, and these differences are independent of sex hormones [17]. This study aims to investigate the role and mechanism of the newly discovered lncRNA ENSSSCG00000035331 in alleviating hippocampal neuronal ferroptosis after CA and resuscitation. To avoid interference from gender differences in experimental results, using single‐sex adult animals ensures physiological consistency among experimental groups. Selecting adult male pigs and mice effectively reduces variables introduced by gender differences, thereby enhancing the accuracy and reproducibility of the experimental results. Additionally, most of our previous studies have used male animals [18], which helps establish a consistent research foundation and facilitates cross‐study comparisons and comprehensive analysis.

### 2.3 The Establishment of the Pig Model of CA and Resuscitation

Initially, the pig was sedated with a mixture of intramuscular thiamylal/zolazepam (5 mg/kg) and xylazine (1 mg/kg), followed by induction of anesthesia with intravenous propofol (2 mg/kg) and then its continuous maintenance at 4 mg/kg/h. Subsequently, endotracheal intubation was performed, and a ventilator (SV350, Mindray, Shenzhen, China) was programmed with the following ventilation parameters: tidal volume of 10 mL/kg, respiratory frequency of 12 breaths/min, peak flow rate of 40 L/min, and a fractional inspired oxygen concentration of 0.21. Concurrently, the levels of end‐tidal CO_2_ were monitored using an integrated defibrillator/monitor (M Series, ZOLL Medical Corporation, Chelmsford, America). The monitoring of hemodynamics, including heart rate and arterial and atrial pressures, was achieved using a patient monitoring system (BeneVision N15, Mindray, Shenzhen, China), in which adhesive electrodes adhered to the skin of the upper and lower limbs, and two 7 Fr pressure‐monitoring catheters were advanced from the right femoral artery and vein into the thoracic aorta and right atrium. CA was induced by navigating a 5F pacing catheter through the right external jugular vein into the right ventricle. Baseline arterial blood gas was measured using a blood gas analyzer (i15, Edan, Shenzhen, China).

After the completion of all surgical preparation and baseline measurements, CA was electrically induced and then untreated for a continuous 10‐min duration. After that, CPR was begun with a 30:2 compression‐to‐ventilation ratio. The quality of chest compression was stably maintained with the help of a real‐time CPR feedback device (PlamCPR, Shangling, Suzhou, China). At 2 min after CPR, epinephrine was intravenously administered at a dose of 20 μg/kg, followed by a repeated dose every 3 min. After 6 min of CPR, a 150‐J biphasic shock was delivered via the defibrillator/monitor. This procedure was rigorously carried out until return of spontaneous circulation (ROSC) was achieved or for a maximum of 16 min. After resuscitation, the animals were closely monitored for 4 h and then returned to their cages for a 20‐h extended observation phase. At 24 h after resuscitation, two independent and blinded investigators evaluated the neurological deficit score (NDS) and cerebral performance category (CPC) [19], which employed the weighted scoring system to assess the degree of neurological dysfunction. The score of NDS ranged from 0 (denoting the absence of neurological deficit) to 400 (signifying death or brain death), and the score of CPC ranged from 1 to 5 (indicating normal cerebral function, moderate cerebral dysfunction with reduced consciousness, severe cerebral disability with significant consciousness impairment, coma or a persistent vegetative state, and death, respectively).

### 2.4 The Establishment of the Mouse Model of CA and Resuscitation

Initially, the mouse was anesthetized with an intraabdominal injection of pentobarbital sodium (45 mg/kg). The electrocardiogram was continuously measured and recorded by a BL‐420N data acquisition and analysis system (Techman, Chengdu, China). Subsequently, endotracheal intubation was performed with a 20‐G catheter, the ventilation was implemented using a ventilator (ALCOTT, Shanghai, China) with an oxygen concentration of 0.21, and then a PE‐10 catheter was inserted into the right jugular vein for drug delivery. During animal preparation, body temperature was maintained at 37.0°C ± 0.5°C with the help of a temperature‐regulated heating pad (RWD, Shenzhen, China). After that, the animal was stabilized for 10 min, and then a dose of 50 μL of KCl (0.5 mol/L) was rapidly infused into the jugular vein to induce CA. After 8 min of CA, mechanical ventilation was resumed with an oxygen concentration of 1.0, a dose of 0.1 mL of epinephrine (16 μg/mL) was administered, and meanwhile finger chest compressions were performed at a rate of approximately 300 compressions per minute. Additional doses of epinephrine were given every 1 min until ROSC was achieved. The animal that failed to achieve ROSC within 5 min or could not be weaned from the ventilator at 1 h of observation was excluded from the experiment. After resuscitation, the animal was observed for a total of 24 h. Thereafter, two independent and blinded investigators conducted the evaluation of neurological function using two neurological function scoring (NFS) systems, in which NFS‐1 was obtained according to the levels of consciousness, respiration, corneal reflex, coordination, and movement, and ranged from 0 (coma) to 10 (fully alert); NFS‐2 was obtained according to the levels of consciousness, respiration, corneal reflex, coordination, movement, and righting reflex, and ranged from 0 (coma) to 12 (fully alert).

### 2.5 LncRNA‐Seq

LncRNA‐seq was performed using the fresh hippocampal tissue samples harvested from the Sham and CA/CPR groups. Briefly, libraries were constructed using 3 μg of RNA from each sample. We followed the protocols of the NEBNext Ultra RNA Library Prep kit for Illumina (New England Biolabs, Ipswich, America). We used the AMPure XP system (Beckman Coulter, Brea, America) to purify the fragments, and those fragments with lengths of 150–500 bp were selected. The cDNA was then digested with USER enzyme before conducting polymerase chain reaction (PCR). Purification of the PCR products and the clustering of the index‐coded samples were performed on the Agilent Bioanalyzer 2100 system (Agilent Technologies, Santa Clara, America) and checked using RNase‐free agarose gel electrophoresis. After total RNA was extracted, rRNAs were removed to retain mRNAs and ncRNAs. The enriched mRNAs and ncRNAs were fragmented into short fragments by using fragmentation buffer and reverse transcribed into cDNA with random primers, second cDNA was synthesized by DNA polymerase I, RNase H, dNTP (dUTP instead of dTTP), and buffer. Next, the cDNA fragments were purified with a QiaQuick PCR extraction kit (Qiagen, Venlo, Netherlands), end‐repaired, poly(A) added, and ligated to Illumina sequencing adapters. Then Uracil‐N‐Glycosylase was used to digest the second‐strand cDNA. The digested products were selected by agarose gel electrophoresis, PCR amplified, and sequenced using Illumina novaseq6000. Qualified libraries were pooled and sequenced on an Illumina platform using the PE150 strategy to analyze differentially expressed lncRNAs. Sequenced reads were trimmed for adaptor sequence, and masked for low‐complexity or low‐quality sequence, then mapped to Ensembl_release 100 whole genome using HISAT2 with parameters‐rna‐strandness RF. Quantification of gene expression level: feature Counts v1.5.0‐p3 was used to count the reads numbers mapped to each gene. The TPM of each gene was calculated based on the length of the gene and the reads count mapped to this gene. FPKM and transcripts per million were calculated. Differentially expressed genes were identified using DESeq2 with raw read counts. The potential binding sites between ENSSSCG00000035331 and miR‐let7a, and between miR‐let7a and GPX4 were analyzed using miRanda (http://www.microrna.org/microrna/home.do), PITA (http://genie.weizmann.ac.il/pubs/mir07/mir07_dyn_data.html), and RNAhybrid (http://bibiserv.techfak.uni‐bielefeld.de/rnahybrid/).

### 2.6 Primary Porcine Hippocampal Neuron Culture

Prepared materials included poly‐L‐lysine, complete medium for hippocampal neurons (Zhongqiao Xinzhou, Shanghai, China), ACCUTASE enzyme (Merck, Darmstadt, German), multiple pairs of ophthalmic surgical scissors, several pairs of tweezers, and other necessary cell culture consumables and reagents. The day before, 6‐well plates/T25 flasks were coated with 0.1 mg/mL sterile‐filtered poly‐L‐lysine (Merck, Darmstadt, German), aspirated following overnight incubation for reuse, and rinsed once with sterile water. Newborn piglet within 24 h of birth was disinfected twice with 75% alcohol, then the brain tissue was harvested and rinsed once with phosphate buffer saline (PBS), in which the skull was opened along the midline using upward‐pointing scissors to minimize brain damage and the intact brain was promptly immersed in ice‐cold Dulbecco's modified eagle medium (TMO, Massachusetts, America). Employing fine straight and curved tweezers, the hippocampus was carefully dissected out, ensuring the removal of any adherent vascular membranes, and promptly transferred to a new ice‐cold Dulbecco's modified eagle medium 6‐cm dish. Iris scissors were used to section the hippocampus into tissue blocks of approximately 1 mm^3^. Sedimentation or centrifugation at 500 rpm for 3 min was allowed, the supernatant was aspirated, 700 μL of Accutase enzyme was added, and digestion occurred at 37°C for 15 min with shaking every 5 min. Following digestion, 2 mL of culture medium was added, pipetting was performed to completely disperse the tissue until no visible tissue blocks remained, and the supernatant was aspirated into a 15‐mL centrifuge tube. Centrifugation at 1000 rpm for 5 min ensued, the supernatant was aspirated, and the cells were resuspended in a complete medium. The prepared cells were transferred to a 37°C CO_2_ incubator for cultivation, and thereafter, half of the medium was replaced every 3 d.

### 2.7 Enzyme‐Linked Immunosorbent Assay

Fresh serum samples were collected and applied to enzyme‐labeled plates precoated with neuron‐specific enolase (NSE) and S100β antibodies (Meixuan Biotechnology Inc., Shanghai, China), ensuring specific binding of NSE and S100β to their respective immobilized antibodies within the enzyme‐linked immunosorbent system. The optical density values at 450 nm were recorded for each well using an ELISA reader (Multiskan SkyHigh, Massachusetts, America), and the concentrations of NSE and S100β in the samples were determined by interpolating these values on a standard curve.

### 2.8 Hematoxylin Eosin Staining

Hippocampal and cortical tissues were promptly immersed in formaldehyde (Beyotime, Shanghai, China) for fixation upon acquisition to inhibit autolysis and putrefaction, and thus preserved the structural integrity of cells and tissues. After that, dehydration was performed using an ethanol (Sinopharm, Sichuan, China) gradient, succeeded by xylene (Sinopharm, Sichuan, China) transparency to enhance paraffin infiltration. Next, the transparent tissue was embedded in paraffin blocks, sectioned to a thickness of 5 μm (Leica Biosystems, Wetzlar, German), dried, dewaxed, and rehydrated. Hematoxylin staining (Beyotime, Shanghai, China) which stained cell nuclei deep blue, was applied, followed by eosin staining, which imparted a pink or red hue to the cytoplasm. The sections were subjected to alcohol hydration, dehydration, and a second xylene transparency step, then mounted onto glass slides, sealed with resin, and allowed to cure. At this stage, the stained histological sections were ready for microscopic (Leica Biosystems, Wetzlar, German) examination and documentation of hippocampal tissue morphology.

### 2.9 TdT‐Mediated dUTP Nick‐End Labeling Assay

After the sections of hippocampal and cortical tissues were obtained as mentioned above, they were sequentially washed with xylene, absolute ethanol, 95% and 75% ethanol, and PBS to eliminate embedding media. Proteinase K (Beyotime, Shanghai, China) digestion was used to digest tissue proteins, followed by rinsing with distilled water and incubation with PBS containing 2% hydrogen peroxide. After a second PBS wash, excess liquid was removed with filter paper. The TdT enzyme buffer and TdT reaction mixture (Boster Biological Technology co. ltd, Wuhan, China) were then added to incubate at 37°C for 1 h. The sections were next transferred to pre‐warmed washing and termination reaction buffer and incubated at 37°C for 30 min with gentle agitation every 10 min. A final PBS wash was conducted before the application of the anti‐digoxigenin antibody, and then incubated at room temperature for 30 min. The sections were then subjected to PBS washing, followed by DAB chromogen incubation for 3–6 min to allow color development. Distilled water washing was followed by counterstaining with methyl green for 10 min. The sections were subsequently dehydrated through ascending grades of distilled water, n‐butanol, and xylene, and finally cover‐slipped, dried, and microscopically evaluated (Biological microscope CX31, Olympus, Japan) for result documentation.

### 2.10 Immunofluorescence Staining

Fresh hippocampal tissues were rapidly frozen with liquid nitrogen and cryosectioned at a thickness of 10 μm. In accordance with the ROS detection kit (share‐bio, shanghai, China), the DCFH‐DA ROS fluorescence probe was diluted 200–1000‐fold with pure water to formulate a staining working solution. At room temperature, 200 μL of the prepared washing solution was carefully applied to fully cover the cryosection and left to stand for 5–10 min. The washing solution was subsequently removed by careful aspiration. Subsequently, 100 μL of the staining working solution was applied, and the sections were incubated in a dark, 37°C incubator for 20–60 min. The staining solution was then removed, and the sections were washed two or three times with PBS. Lastly, the sections were covered with a coverslip or glycerol mounting medium and subjected to fluorescent microscopy (Biological microscope CX31, Olympus, Japan) for visualization.

### 2.11 qRT‐PCR

Total RNA from primary porcine hippocampal neurons, porcine, and mouse hippocampus tissues was extracted using TRIzol reagent (Invitrogen, Carlsbad, America). RNA quantification was then performed using a Nanodrop instrument (TMO, Massachusetts, America). Subsequently, cDNA synthesis was carried out using an RT kit (Yeasen, Shanghai, China). PCR amplification was conducted using qPCR Mix (Yeasen, Shanghai, China). Relative expression levels were calculated using the 2^−ΔΔCt^ method. The primers utilized in this study were provided in the Table S1.

### 2.12 Fe^2+^ Detection

Following the instructions of the bc5415 Fe^2+^ detection kit (Solarbio, Beijing, China), fresh hippocampal tissues or primary porcine hippocampal neurons were procured. The samples were homogenized under ice‐bath conditions and then centrifuged to collect the supernatant. Prepare standard solutions and dilute them into a series of concentrations. Aliquot each concentration of the diluted standards and sequentially introduce Reagent II and chloroform (Sinopharm, Sichuan, China). Measure the absorbance at 593 nm for each standard, and plot a standard curve correlating absorbance values with their respective concentrations. Repeat the above procedures on the supernatant obtained from the test porcine hippocampal tissue or primary porcine hippocampal neurons. Calculate the corrected absorbance ΔA as the difference between A_measured (absorbance of the test sample) and A_blank (absorbance of control without the sample). Utilize the established standard curve to determine the sample concentration (x, μmol/L) by substituting ΔA (y, ΔA) into the relevant formula.

### 2.13 GSH Assay

Employ a microplate‐based GSH assay kit (Nanjing Jiancheng Bioengineering Institute, Nanjing, China), and homogenize those freshly harvested hippocampal tissues or primary porcine hippocampal neurons in physiological saline at a defined ratio. Centrifugation ensued, and the supernatant was harvested. A series of standard solutions were prepared by diluting the stock solution to multiple concentrations. Absorbance readings at a wavelength of 405 nm were taken for each concentration, and a standard curve was constructed accordingly. The identical procedure was repeated on the supernatant obtained from the samples of hippocampal tissues or primary porcine hippocampal neurons. The absorbance change (ΔA) was computed as ΔA = A_measured_—A_blank_. Utilizing the established standard curve, the sample concentration was derived by incorporating the obtained ΔA value into the relevant formula.

### 2.14 Fluorescence In Situ Hybridization (FISH)

The enhanced sensitive ISH detection kit V (FITC) for FISH (Boster, Wuhan, China) was utilized for FISH probe labeling of ENSSSCG00000035331 according to the manufacturer's guidelines and recommendations. Briefly, hippocampal paraffin sections were conventionally deparaffinized until reaching water and succeeded by room temperature digestion to expose mRNA molecules. Subsequently, the sections were incubated in a hybridization prebuffer (37°C, 30 min), and followed by overnight exposure in a room temperature hybridization buffer containing a 20 μM lncRNA FISH probe mixture. The specimens were then washed in 0.1% Tween‐20 solutions of 2 × SSC, 0.5 × SSC, and 0.2 × SSC, each for 15 min. The sections were subsequently blocked at 37°C for 30 min, followed by the addition of biotinylated mouse anti‐digoxigenin, SABC‐FITC, and DAPI for nuclear staining. Finally, following mounting with an anti‐fade mounting medium, the slides were visualized under a fluorescence microscope (Biological microscope CX31, Olympus, Japan). Probe sequence: 5′GGTTTCACTTGGATTGGTTGCTGTAAGACGCACTGAAGCCAA'3‐FITC.

### 2.15 Construction of ENSSSCG00000035331 Overexpression Vector and Establishment of H/R Model of Primary Porcine Hippocampal Neurons

The overexpression construct of ENSSSCG00000035331 was separately integrated into adeno‐associated viruses, along with the construction of a negative control adeno‐associated viruses, all of which were carried out by Quanyang Biotechnology (Shanghai, China). To begin, primary porcine hippocampal neurons were seeded at a density of 5 × 10^5^ cells per well into 6‐well plates. Following the transfection for 48 h, primary neurons were treated with glucose‐free Dulbecco's modified eagle medium and placed within an incubator simulating hypoxic conditions, filled with a gas mixture composed of 1% oxygen, 5% carbon dioxide, and 94% nitrogen, maintained at 37°C for 3 h to induce hypoxic injury. After that, the culture medium was replaced with a fresh neuronal basal medium, and then the neurons were subjected to reperfusion culture for an additional 24 h under normoxic conditions. In contrast, control group neurons were always cultured under normal conditions in a standard incubator.

### 2.16 miR‐let7a Regulation Experiment in Primary Porcine Hippocampal Neuron

miR‐let7a mimics and miR‐let7a inhibitors were constructed by Quanyang Biotechnology (Shanghai, China), and then stored and diluted according to the manufacturer's instructions. Briefly, porcine hippocampal neurons were seeded onto 6‐well plates at a density of 5 × 10^5^ cells per well, and both miRNA and Lipofectamine 3000 (TMO, Massachusetts, America) were individually diluted in RNase‐free water or Opti‐MEM medium (TMO, Massachusetts, America). They were then mixed at the recommended ratio provided by the manufacturer to form miRNA transfection complexes. These complexes were added to the cell culture plates, gently rocking the plates to ensure even distribution of the complexes over the cells. After a 48‐h transfection period, the cells were subjected to further experiments.

### 2.17 Cell Viability and Lactate Dehydrogenase (LDH)

Cell viability was measured with the CCK‐8 assay kit (Biyuntian Biotechnology, Shanghai, China) in the 96‐well plate. After 10 μL of CCK‐8 reagent was added to each well to incubate for 3 h at 37°C, and then the absorbance was measured at 450 nm with the microplate reader (Multiskan MK3, Thermo Fisher Scientific, Waltham, MA). LDH release analysis was performed with the LDH assay kit (Abcam, Cambridge, MA). After the cell supernatant was collected and mixed with the LDH assay buffer, and then they were incubated at 37°C for 1 h. The mixture was measured at 450 nm with the help of the microplate reader above.

### 2.18 Luciferase Reporter Assay

To confirm the binding between ENSSSCG00000035331 and miR‐let7a, the wild‐type or mutated ENSSSCG00000035331 sequences were cloned into the psiCHECK2.0 vector (TongYong Bio, Anhui, China), and HEK‐293T cells were then transiently co‐transfected by the psiCHECK2.0 plasmids with either miR‐let7a mimics or negative controls. After that, the activities of Firefly and Renilla luciferase were assessed using the Dual‐Luciferase Reporter Assay System (Promega, Madison, America) according to the manufacturer's instructions, then measured with a fluorescence spectrophotometer (Tecan, Männedorf, Switzerland), and finally the relative ENSSSCG00000035331 Firefly luciferase activity was normalized to Renilla luciferase activity. To confirm the binding between miR‐let7a and GPX4, the wild‐type or mutated GPX4‐3'‐UTR sequences were cloned into the psiCHECK2.0 vector (TongYong Bio, Anhui, China), HEK‐293T cells were similarly co‐transfected, then the activities of Firefly and Renilla luciferase were similarly assessed and measured, and finally the relative GPX4 Firefly luciferase activity was normalized to Renilla luciferase activity.

### 2.19 RNA Pull‐Down Assay

To confirm the interaction between ENSSSCG00000035331 and miR‐let7a, biotin‐labeled ENSSSCG00000035331 or normal control (NC) (TongYong Bio, Anhui, China) was constructed according to the manufacturer's instructions. Firstly, the H/R model of hippocampal neurons was established. Subsequently, the cells were incubated with lysis buffer for 10 min, and then cell lysates were incubated with biotin‐labeled ENSSSCG00000035331 or NC at 4°C overnight. After that, they were combined with M‐280 streptavidin beads to incubate at 4°C for 3 h, followed by washing with low‐salt buffer for 3 times. Finally, the enrichment of miR‐let7a was detected by PCR analysis. To confirm the interaction between miR‐let7a and GPX4, biotin‐labeled GPX4 or NC (TongYong Bio, Anhui, China) were constructed according to the manufacturer's instructions. Similarly, the H/R model of hippocampal neurons was established, and then the cells were lysed and incubated with biotin‐labeled GPX4 or NC, thereafter combined with streptavidin beads and washed, and finally detected for miR‐let7a enrichment using PCR analysis. The biotin‐labeled probe sequences used in this study are listed in Table S2.

### 2.20 Western Blot

Fresh samples of hippocampal tissues or primary porcine hippocampal neurons were collected and lysed using the RIPA lysis buffer (Beyotime, Shanghai, China). Protein samples (20 μg/well) were loaded onto pre‐cast 15% SDS‐PAGE gels (GenScript, Nanjing, China). Subsequently, proteins were transferred onto polyvinylidene difluoride membranes (Millipore, Massachusetts, America). Membranes were then blocked with 5% BSA (Beyotime, Shanghai, China), incubated with primary antibodies, and followed by secondary antibodies. Finally, protein bands were visualized using an ECL detection kit (Millipore, Massachusetts, America) and analyzed using ImageJ software (NIH, America). The antibodies used included: anti‐GPX4 antibody (diluted in 1:1000; Abcam, America), anti‐GAPDH antibody (diluted in 1:2000; Abcam, America), and goat anti‐rabbit IgG (H + L) HRP‐conjugated secondary antibody (diluted in 1:10000; ZSGB, China).

### 2.21 Flow Cytometry

Porcine hippocampal neurons were digested, collected, washed three times with PBS, and centrifuged (1000 g, 5 min) to form pellets. Resuspend the pellets using Annexin V (share‐bio, shanghai, China), ROS (share‐bio, shanghai, China), or BODIPY 581/591 C11 fluorescent probes (MCE, New Jersey, America), followed by incubation at room temperature in the dark for 10–20 min. Subsequently, rewash the samples to remove excess fluorescent probes, and finally place them on ice in a 4°C bath. Following propidium iodide signal detection, the cell cycle was analyzed utilizing the CytoFLEX flow cytometer (Dalewe, Shenzhen, China).

### 2.22 Statistical Analysis

Statistical analysis was performed using a random blind method. Before comparisons, all datasets underwent Shapiro–Wilk or Kolmogorov–Smirnov tests to assess whether they met the assumption of normal distribution. For those data that conformed to a normal distribution, they were expressed as mean ± standard deviation, and Student's *t*‐test was employed for statistical comparisons between two groups, and one‐way ANOVA and Tukey's multiple comparison tests were utilized to evaluate the differences among multiple groups (using GraphPad Prism version 8.3.0). When the data were not normally distributed, they were expressed as a median (25th, 75th percentiles), and then the Kruskal–Wallis test was used to analyze these nonparametric data. Statistical significance was set at *p* < 0.05.

## 3 Results

### 3.1 Brain Injury Was Observed in a Pig Model of CA and Resuscitation

**FIGURE 1:**
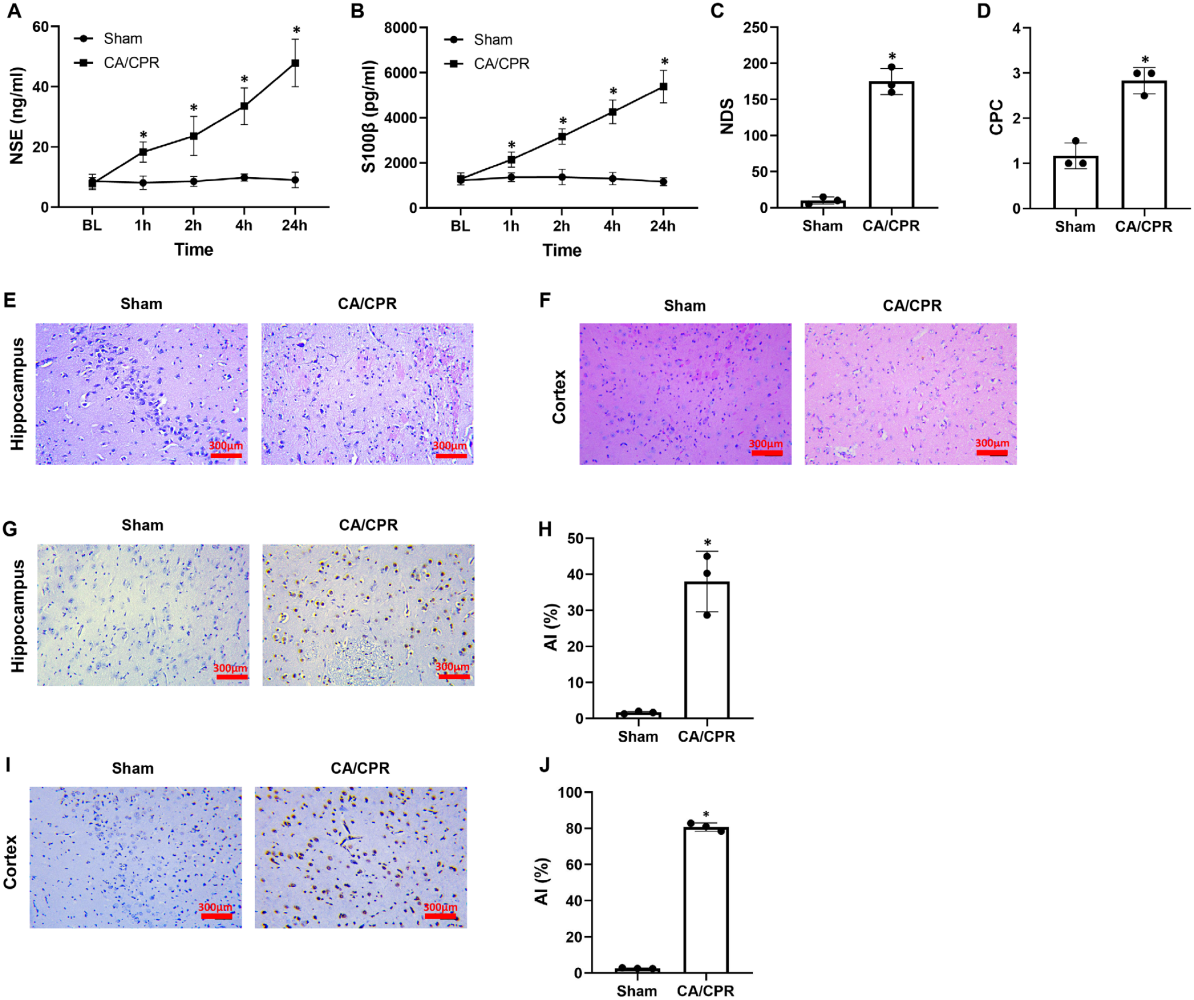
Brain injury occurred after cardiac arrest (CA) and resuscitation in pigs. (A, B) Changes of serum concentrations of neuron‐specific enolase (NSE) and S100β protein (S100β) at baseline (BL), and 1 h, 2 h, 4 h, and 24 h post‐resuscitation. (C, D) Evaluation of neurological function using neurological deficit score (NDS) and cerebral performance category (CPC) at 24 h post‐resuscitation. (E, F) Representative photographs of hematoxylin and eosin staining in hippocampal and cortical tissues at 24 h post‐resuscitation (Scale bar = 300 μm, ×200 magnification). (G–J) Representative photographs of TdT‐mediated dUTP nick‐end labeling staining in hippocampal and cortical tissues at 24 h post‐resuscitation (Scale bar = 300 μm, ×200 magnification) and their apoptotic index (AI). CPR, cardiopulmonary resuscitation. Each group included three samples. *p < 0.05 denotes significant differences compared to the Sham group.

To recreate brain injury after CA and resuscitation, three pigs were used to establish the pig model using the setting of 10 min of CA and 6 min of CPR. Those physiological indicators (body weight, heart rate, mean arterial pressure, and end‐tidal CO_2_), arterial blood gas (pH, PCO_2_, PO_2_, and lactate), and brain injury biomarkers (NSE, S100β) at baseline were not different between the Sham and CA/CPR groups (Figure S1A–H, Figure 1A,B). During the model establishment, coronary perfusion pressure was regularly monitored and the outcomes of CPR including duration of CPR, dosage of epinephrine, and number of defibrillations were recorded in the CA/CPR group, in which similar results were observed among the three pigs. Finally, all three pigs obtained ROSC (Figure S1I–M). After resuscitation, the serum levels of NSE and S100β were significantly increased at all time points, and the scores of NDS and CPC at 24 h were markedly elevated in the CA/CPR group when compared with the Sham group (Figure 1A–D). Furthermore, hippocampal and cortical tissue analysis indicated that pathological injury including tissue structure disarrangement, cell integrity damage, nucleus pycnosis, and inflammatory cell infiltration was obvious and the ratio of cell apoptosis was significantly higher in the CA/CPR group when compared to the Sham group (Figure 1E–J). These results indicated that post‐resuscitation brain injury was successfully recreated in this pig model of CA and resuscitation.

### 3.2 The Phenomenon of Cell Ferroptosis Occurred in the Impaired Hippocampus After CA and Resuscitation in Pigs

**FIGURE 2:**
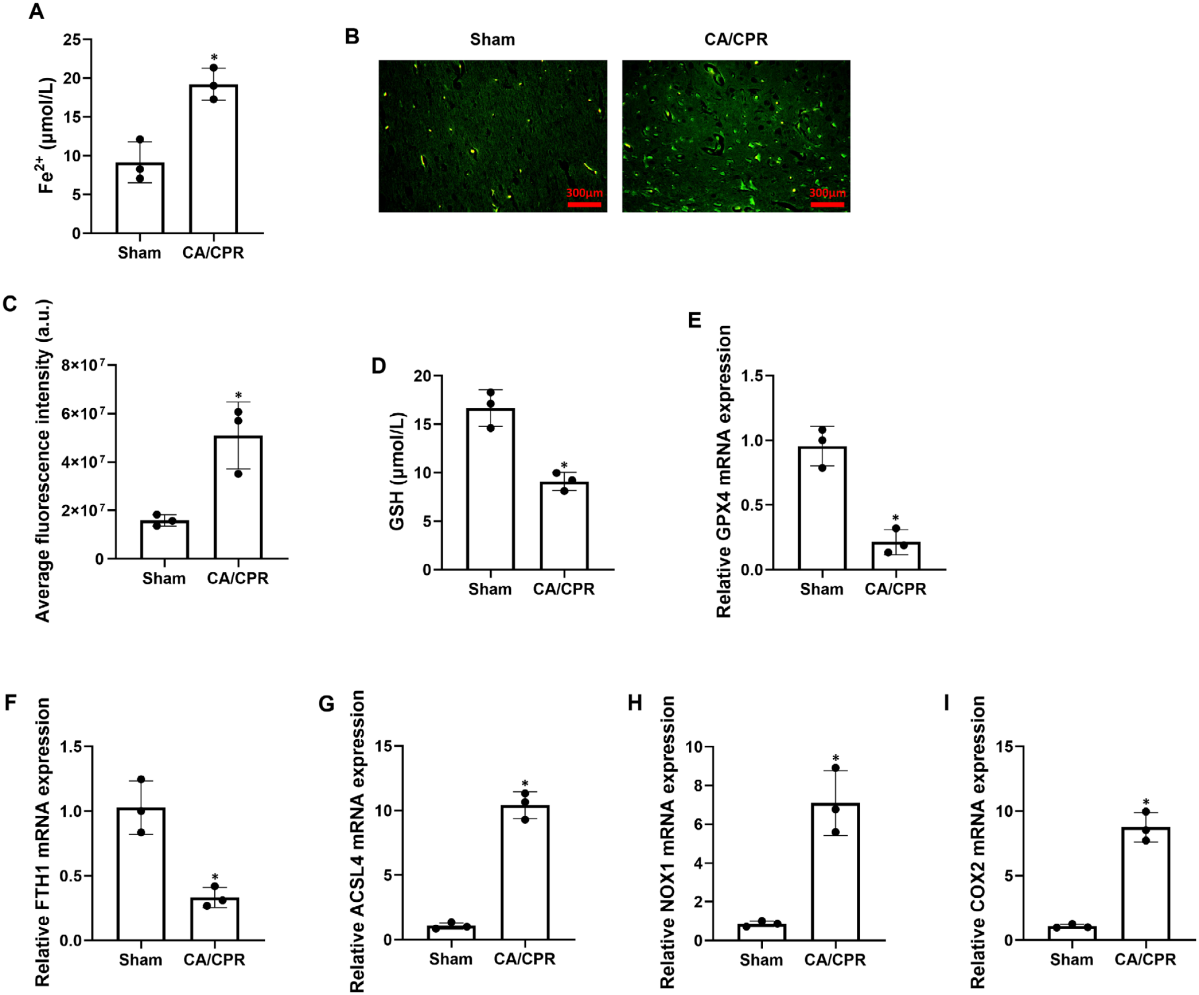
Ferroptosis was evident in the impaired hippocampus after cardiac arrest (CA) and resuscitation in pigs. (A) Levels of ferrous iron (Fe^2+^) in hippocampal tissues at 24 h post‐resuscitation. (B, C) Representative photographs of immunofluorescence staining of reactive oxygen species production in hippocampal tissues at 24 h post‐resuscitation (scale bar = 300 μm, ×200 magnification) and its quantification. (D) Glutathione (GSH) contents in hippocampal tissues at 24 h post‐resuscitation. (E–I) Relative mRNA expression levels of ferroptosis‐associated genes including glutathione peroxidase 4 (GPX4) (E), ferritin heavy chain 1 (FTH1) (F), acyl‐CoA synthetase long‐chain family member 4 (ACSL4) (G), NADPH oxidase 1 (NOX1) (H), and cyclooxygenase‐2 (COX2) (I) in hippocampal tissues at 24 h post‐resuscitation. CPR, cardiopulmonary resuscitation. Each group included three samples. *p < 0.05 denotes significant differences compared to the Sham group.

To confirm that cell ferroptosis was involved in the pathological process of brain injury after CA and resuscitation, the phenomenon of cell ferroptosis was evaluated in the post‐resuscitation impaired hippocampus in this pig model. First, those productions related to ferroptosis, including Fe^2+^, ROS, and GSH, were measured. We observed that Fe^2+^ levels and ROS production were significantly increased while GSH contents were significantly decreased in the post‐resuscitation hippocampus in the CA/CPR group when compared with the Sham group (Figure 2A–D). Second, those key genes related to ferroptosis, including the antioxidant defense gene GPX4, iron storage protein‐encoding gene ferritin heavy chain 1 (FTH1), lipid metabolism‐related gene acyl‐CoA synthetase long‐chain family member 4 (ACSL4), ROS‐producing enzyme NADPH oxidase 1 (NOX1), and inflammation‐related gene cyclooxygenase‐2 (COX2) were measured. We observed that the relative expression levels of GPX4 and FTH1 mRNA were significantly decreased while the relative expression levels of ACSL4, NOX1, and COX2 mRNA were significantly increased in the post‐resuscitation hippocampus in the CA/CPR group when compared to the Sham group (Figure 2E–I). These results indicated that the phenomenon of cell ferroptosis occurred in the post‐resuscitation impaired hippocampus, which might participate in the pathological process of brain injury after CA and resuscitation in pigs.

### 3.3 ENSSSCG00000035331 Overexpression Promoted Cell Survival and Inhibited Its Ferroptosis After H/R Stimulation in Primary Porcine Hippocampal Neurons

**FIGURE 3:**
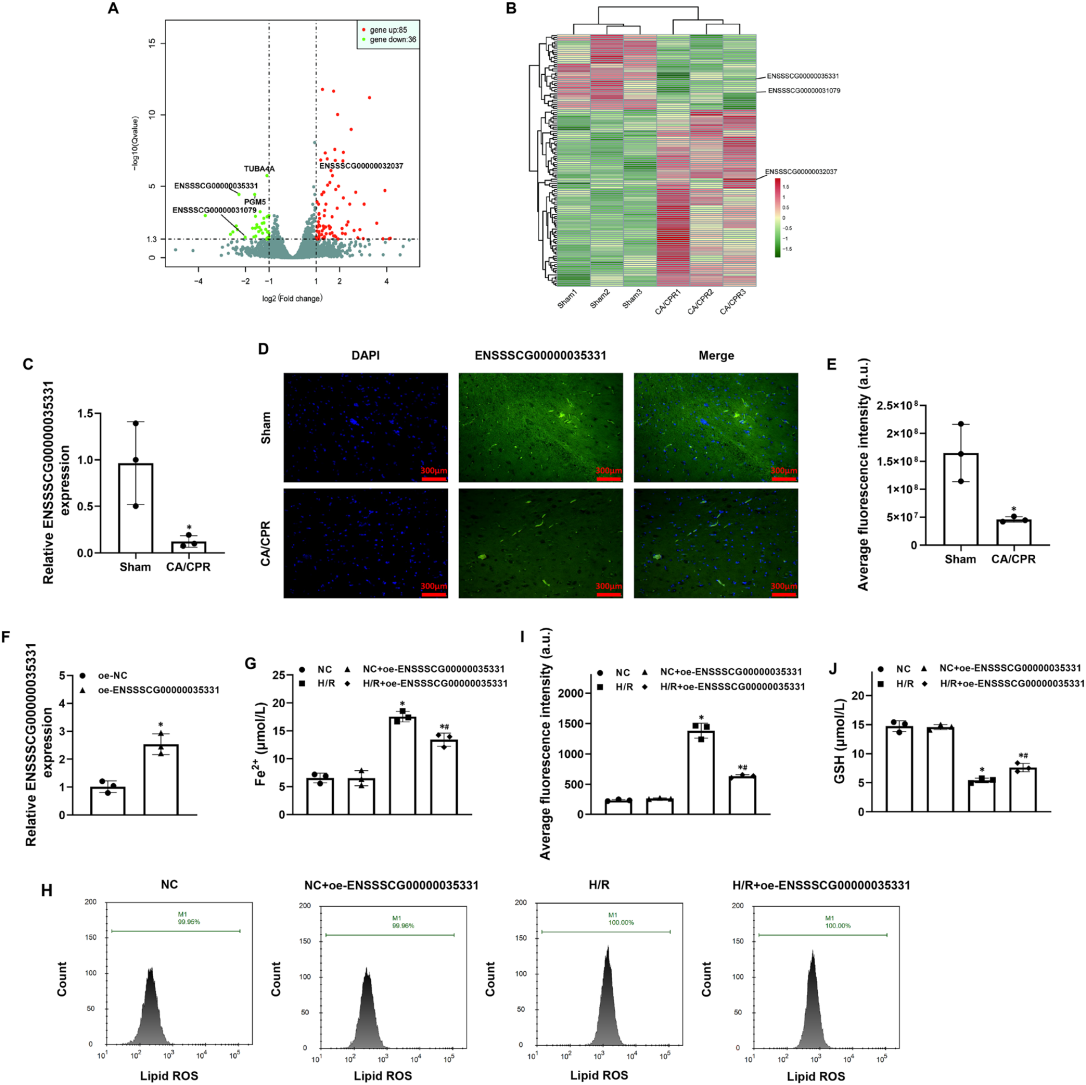
LncRNA ENSSSCG00000035331 overexpression alleviated hippocampal neuronal ferroptosis after hypoxia/reoxygenation (H/R) stimulation. (A) Volcano plot representing differentially expressed genes in hippocampal tissues at 24 h post‐resuscitation between the cardiac arrest and cardiopulmonary resuscitation (CA/CPR) and Sham groups. Red color indicates that those genes were significantly upregulated, green color indicates that those genes were significantly downregulated, and gray color indicates that those genes' expression weren't changed. (B) Heatmap plot analysis of the differentially expressed genes identified in (A), which indicates that lncRNAs ENSSSCG00000035331 and ENSSSCG00000031079 were significantly downregulated, and lncRNA ENSSSCG00000032307 was significantly upregulated in the CA/CPR group compared with the Sham group. (C) The comparative analysis of relative expression levels of ENSSCG00000035331 in hippocampal tissues at 24 h post‐resuscitation between the CA/CPR and Sham groups. (D) Fluorescence in situ hybridization showing the localization of ENSSCG00000035331 in hippocampal tissues at 24 h post‐resuscitation (Scale bar = 300 μm, ×200 magnification). (E) Quantitative analysis of average fluorescent intensity of ENSSCG00000035331 in (D). (F) Confirmation of ENSSCG00000035331 overexpression in primary porcine hippocampal neurons after the transfection of adeno‐associated viruses. (G) Levels of ferrous iron (Fe^2+^) in hippocampal neurons at 24 h after H/R stimulation. (H) Flow cytometric analysis and quantification of lipid reactive oxygen species (ROS) production in hippocampal neurons at 24 h after H/R stimulation. (I) Glutathione (GSH) contents in hippocampal neurons at 24 h after H/R stimulation. NC, normal control. Each group included three samples in animal experiments and three replicates in cell experiments, respectively. *p < 0.05 denotes significant differences compared to the Sham group or the NC group; ^#^ *p* < 0.05 denotes significant differences compared to the H/R group.

To explore the potential key lncRNA regulating post‐resuscitation hippocampal neuronal damage, we employed lncRNA‐Seq technology to conduct deep sequencing, systematic analysis, and experimental verification in the post‐resuscitation impaired hippocampus. The results showed that 85 genes were upregulated and meanwhile 36 genes were downregulated in the CA/CPR group when compared with the Sham group (Figure 3A,B). Among these, six top differentially expressed lncRNAs including ENSSSCG00000035331, ENSSSCG00000031079, ENSSSCG00000040228, ENSSSCG00000032037, ENSSSCG00000034990, and ENSSSCG00000038924 were verified using qRT‐PCR, in which the expression level of ENSSSCG00000035331 was confirmed to be significantly decreased in the post‐resuscitation impaired hippocampus in the CA/CPR group when compared to the Sham group (Figure 3C, Figure S2A–E). Furthermore, the same result of ENSSSCG00000035331 expression was observed using FISH detection, and its location was confirmed to be primarily in the cytoplasm (Figure 3D,E).

To investigate the role of ENSSSCG00000035331 in hippocampal neuronal damage after H/R stimulation, ENSSSCG00000035331 overexpression was constructed in hippocampal neurons using adenoviral transduction and then confirmed using fluorescence and qRT‐PCR (Figure S3A,B, Figure 3F). Subsequently, the effects of ENSSSCG00000035331 overexpression on cell viability, LDH release, apoptosis, and cytosolic ROS production were evaluated in hippocampal neurons after H/R stimulation. We observed that H/R stimulation significantly decreased cell viability while significantly increased LDH release, apoptosis ratio, and cytosolic ROS production in hippocampal neurons; however, all the changes were significantly reversed in the H/*R* + oe‐ENSSSCG00000035331 group when compared with the H/R group (Figure S4A–F). These results indicated that ENSSSCG00000035331 overexpression could promote hippocampal neuronal survival after H/R stimulation.

To further investigate the role of ENSSSCG00000035331 in regulating hippocampal neuronal ferroptosis after H/R stimulation, ENSSSCG00000035331 overexpression was similarly constructed in hippocampal neurons, and then those ferroptosis‐related productions including Fe^2+^, lipid ROS, and GSH were measured after H/R stimulation. We observed that H/R stimulation significantly increased Fe^2+^ levels and lipid ROS production while significantly decreased GSH contents in hippocampal neurons; however, ENSSSCG00000035331 overexpression significantly decreased Fe^2+^ levels and lipid ROS production and meanwhile increased GSH contents in hippocampal neurons in the H/*R* + oe‐ENSSSCG00000035331 group when compared to the H/R group (Figure 3G–J). These results indicated that ENSSSCG00000035331 overexpression could inhibit hippocampal neuronal ferroptosis after H/R stimulation.

### 3.4 miR‐let7a Identified as a Downstream Target of ENSSSCG00000035331 Regulated Hippocampal Neuronal Ferroptosis After H/R Stimulation

**FIGURE 4:**
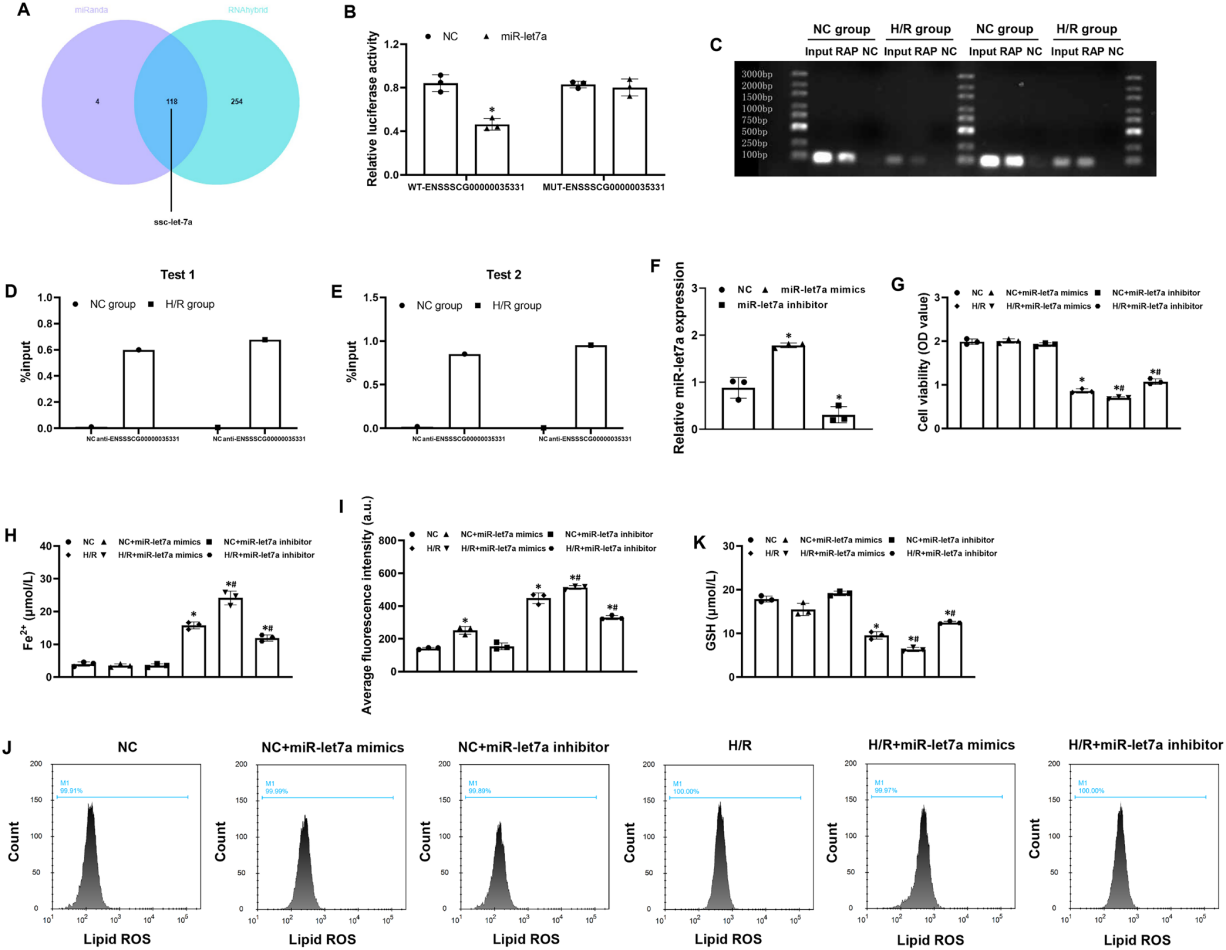
MiR‐let7a was identified as a downstream target of ENSSSCG00000035331 and regulated hippocampal neuronal ferroptosis after hypoxia/reoxygenation (H/R) stimulation. (A) Venn diagram representing miR‐let7a as a potential downstream target of ENSSSCG00000035331 using the miRanda and RNAhybrid software. (B) Luciferase reporter assay and analysis of the binding between ENSSSCG00000035331 and miR‐let7a. (C) RNA pulldown assay of the binding between ENSSSCG00000035331 and miR‐let7a in hippocampal neurons under normal culture conditions and after H/R stimulation. (D, E) Quantitative analysis of miR‐let7a enrichment from two independent RNA pulldown experiments in (C). (F) Relative expression levels of miR‐let7a in primary porcine hippocampal neurons treated with miR‐let7a mimics or miR‐let7a inhibitor. (G, H) Cell viability and ferrous iron (Fe^2+^) levels in hippocampal neurons at 24 h after H/R stimulation. (I, J) Flow cytometric analysis and quantification of lipid reactive oxygen species (ROS) production in hippocampal neurons at 24 h after H/R stimulation. (K) Glutathione (GSH) contents in hippocampal neurons at 24 h after H/R stimulation. NC, normal control. Each group included three replicates. *p < 0.05 denotes significant differences compared to the NC group; ^#^ *p* < 0.05 denotes significant differences compared to the H/R group.

To identify the potential downstream target of ENSSSCG00000035331, a series of bioinformatic analyses and experimental verification were performed. First, the two tools including miRanda and RNAhybrid were used to successfully predict miR‐let7a as a potential downstream target of ENSSSCG00000035331 (Figure 4A). Thereafter, the potential binding between ENSSSCG00000035331 and miR‐let7a was explored using a dual‐luciferase reporter assay and RNA pull‐down assay. Consequently, the dual‐luciferase reporter revealed that relative luciferase activity was significantly decreased by miR‐let7a mimics in ENSSSCG00000035331 wild‐type HEK‐293T cells (Figure 4B). Furthermore, RNA pull‐down revealed that miR‐let7a could bind with ENSSSCG00000035331 in hippocampal neurons under normal culture condition and after H/R stimulation (Figure 4C–E). These results indicated that miR‐let7a was identified as a feasible downstream target of ENSSSCG00000035331 in hippocampal neurons.

To further investigate the role of miR‐let7a in regulating hippocampal neuronal ferroptosis after H/R stimulation, miR‐let7a mimics and miR‐let7a inhibitors were constructed to use in hippocampal neurons, and then the relative expression of miR‐let7a was confirmed using qRT‐PCR (Figure 4F). Subsequently, the effects of miR‐let7a on cell viability and those ferroptosis‐related productions mentioned above were evaluated in hippocampal neurons after H/R stimulation. Similarly, we observed that H/R stimulation significantly decreased cell viability and GSH contents while significantly increased Fe^2+^ levels and lipid ROS production in hippocampal neurons. In addition, miR‐let7a mimics further significantly decreased cell viability and promoted ferroptosis in hippocampal neurons in the H/*R* + miR‐let7a mimics group when compared with the H/R group. However, miR‐let7a inhibitors significantly recovered cell viability and inhibited ferroptosis in hippocampal neurons in the H/*R* + miR‐let7a inhibitor group when compared to the H/R group (Figure 4G–K). These results indicated that miR‐let7a could play a key role in mediating hippocampal neuronal ferroptosis after H/R stimulation.

### 3.5 ENSSSCG00000035331 Inhibited Post‐Resuscitation Hippocampal Neuronal Ferroptosis via Regulating the miR‐let7a/GPX4 Axis

**FIGURE 5:**
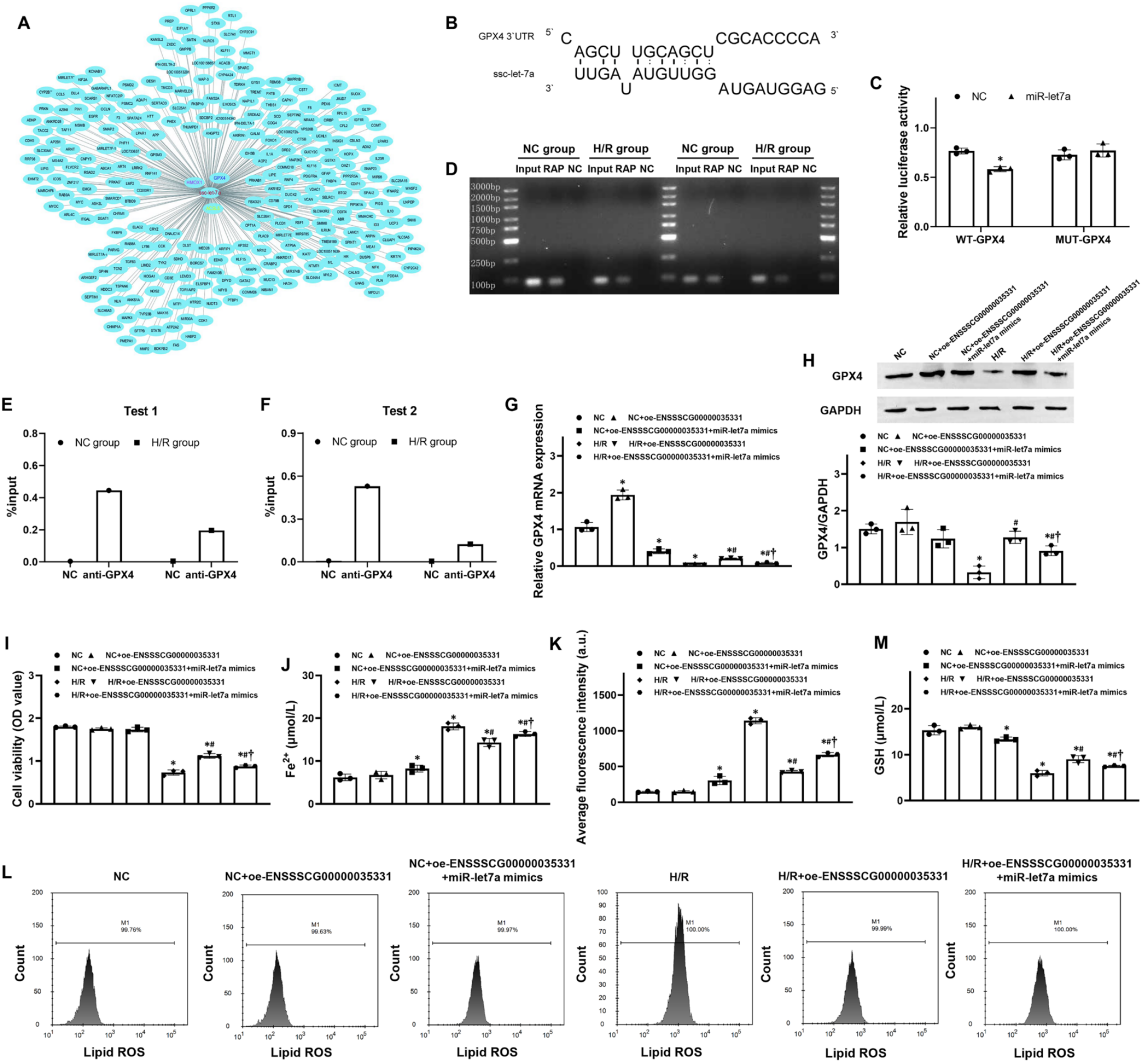
ENSSSCG00000035331 overexpression inhibited hypoxia/reoxygenation (H/R)‐induced hippocampal neuronal ferroptosis via regulating the miR‐let7a/glutathione peroxidase 4 (GPX4) axis. (A) Bioinformatics network analysis illustrating GPX4 as a potential downstream target of miR‐let7a using the miRanda and RNAhybrid software. (B) Diagram showing the binding site of miR‐let7a in the 3′UTR region of the GPX4 gene. (C) Luciferase reporter assay and analysis of the binding between miR‐let7a and GPX4. (D) RNA pulldown assay of the binding between miR‐let7a and GPX4 in hippocampal neurons under normal culture conditions and after H/R stimulation. (E, F) Quantitative analysis of miR‐let7a enrichment from two independent RNA pulldown experiments shown in (D). (G) Relative expression levels of GPX4 mRNA in hippocampal neurons at 24 h after H/R stimulation. (H) Relative expression levels of GPX4 protein in hippocampal neurons at 24 h after H/R stimulation. (I, J) Cell viability and ferrous iron (Fe^2+^) levels in hippocampal neurons at 24 h after H/R stimulation. (K, L) Flow cytometric analysis and quantification of lipid reactive oxygen species (ROS) production in hippocampal neurons at 24 h after H/R stimulation. (M) Glutathione (GSH) contents in hippocampal neurons at 24 h after H/R stimulation. NC, normal control. Each group included three replicates. *p < 0.05 denotes significant differences compared to the NC group; ^#^ *p* < 0.05 denotes significant differences compared to the H/R group; †*p* < 0.05 denotes significant differences compared to the H/*R* + oe‐ENSSSCG00000035331 group.

To explore the potential mechanism by which miR‐let7a regulated hippocampal neuronal ferroptosis after H/R stimulation, miRanda and RNAhybrid were also used to predict the potential downstream target of miR‐let7a, and then their potential binding was similarly explored using dual‐luciferase reporter assay and RNA pull‐down assay. The result showed that GPX4 was one candidate for downstream targets of miR‐let7a, and had the targeted binding site with miR‐let7a (Figure 5A,B). In addition, the dual‐luciferase reporter revealed that relative luciferase activity was significantly decreased by miR‐let7a mimics in GPX4 wild‐type HEK‐293T cells (Figure 5C). RNA pull‐down revealed that miR‐let7a could bind with GPX4 mRNA in hippocampal neurons under normal culture condition and after H/R stimulation (Figure 5D–F). These results indicated that miR‐let7a could bind with GPX4 mRNA, and then inhibit its translation in hippocampal neurons after H/R stimulation [20].

To investigate the role of miR‐let7a in the ENSSSCG00000035331‐induced hippocampal neuronal ferroptosis inhibition after H/R stimulation, ENSSSCG00000035331 overexpression was constructed, and meanwhile, miR‐let7a mimics were used in hippocampal neurons. We observed that H/R stimulation significantly downregulated GPX4 mRNA and protein expression, and decreased GSH contents and cell viability while significantly increased Fe^2+^ levels and lipid ROS production in hippocampal neurons. In addition, ENSSSCG00000035331 overexpression significantly increased GPX4 mRNA and protein expression, recovered cell viability, and inhibited ferroptosis in hippocampal neurons in the H/*R* + oe‐ENSSSCG00000035331 group when compared with the H/R group. However, miR‐let7a mimics significantly abrogated those protective effects produced by ENSSSCG00000035331 overexpression mentioned above in hippocampal neurons in the H/*R* + oe‐ENSSSCG00000035331 + miR‐let7a mimics group when compared to the H/*R* + oe‐ENSSSCG00000035331 group (Figure 5G–M). These results indicated that ENSSSCG00000035331 overexpression could inhibit H/R‐induced hippocampal neuronal ferroptosis by recovering GPX4 mRNA translation via interacting with miR‐let7a.

**FIGURE 6:**
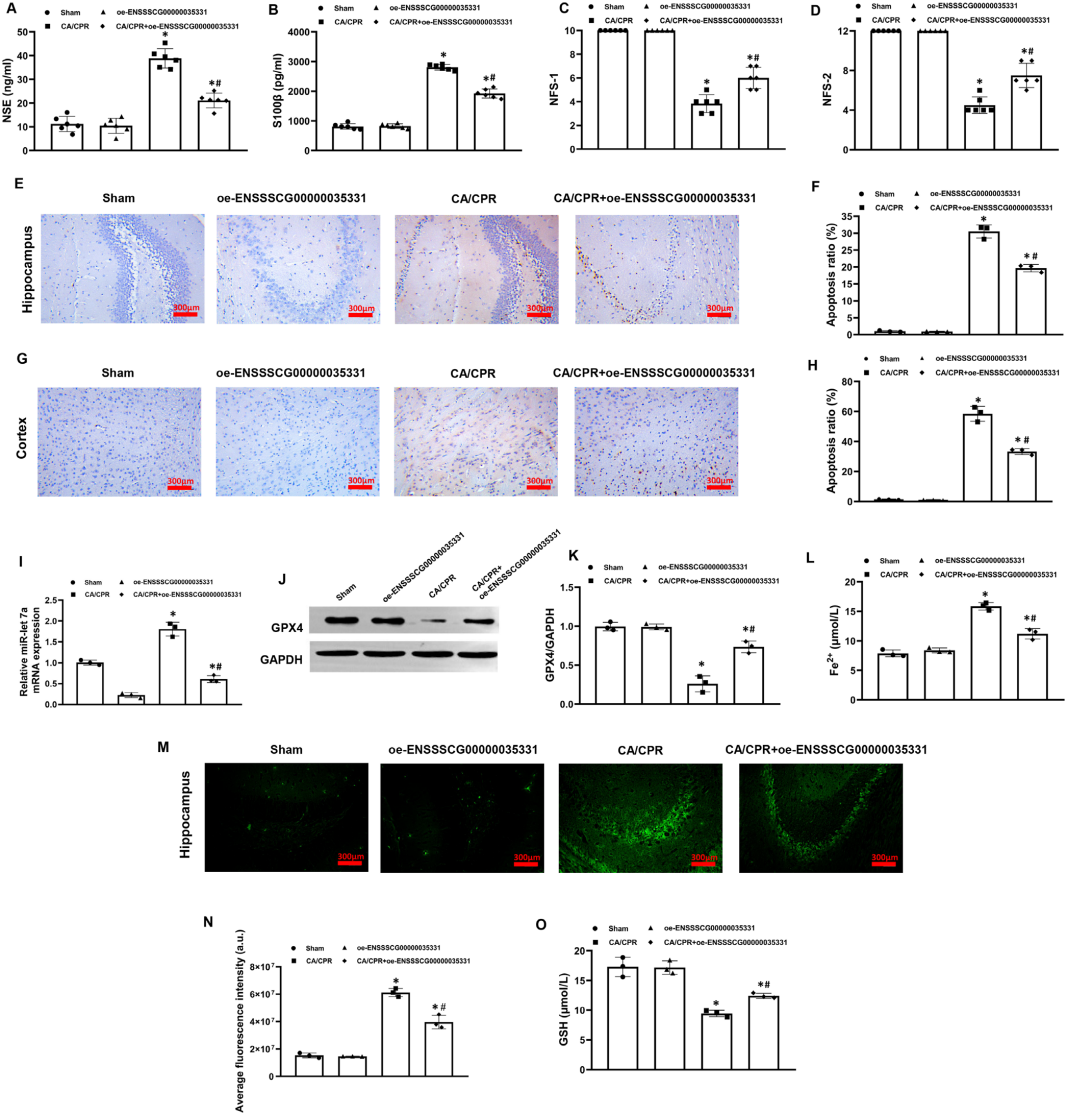
ENSSSCG00000035331 overexpression alleviated post‐resuscitation brain injury and hippocampal neuronal ferroptosis possibly by regulating the miR‐let7a/glutathione peroxidase 4 (GPX4) axis. (A, B) Changes of serum concentrations of neuron‐specific enolase (NSE) and S100β protein (S100β) at 24 h post‐resuscitation. (C, D) Evaluation of neurological function using two methods of neurological functional scores (NFS) at 24 h post‐resuscitation. (E‐H) Representative photographs of TdT‐mediated dUTP nick‐end labeling staining in hippocampal and cortical tissues at 24 h post‐resuscitation (scale bar = 300 μm, ×200 magnification) and its quantification(*n* = 3). (I) Relative miR‐let7a expression levels in hippocampal tissues at 24 h post‐resuscitation. (J, K) Relative expression levels of GPX4 protein in hippocampal tissues at 24 h post‐resuscitation. (L) Levels of ferrous iron (Fe^2+^) in hippocampal tissues at 24 h post‐resuscitation. (M, N) Representative photographs of immunofluorescence staining of reactive oxygen species (ROS) production in hippocampal tissues at 24 h post‐resuscitation (scale bar = 300 μm, ×200 magnification) and its quantification. (O) Glutathione (GSH) contents in hippocampal tissues at 24 h post‐resuscitation. CA, cardiac arrest. CPR, cardiopulmonary resuscitation. Each group included six samples in (A–D) and three samples in (E–O). *p < 0.05 denotes significant differences compared to the Sham group; #*p* < 0.05 denotes significant differences compared to the CA/CPR group.

**FIGURE 7:**
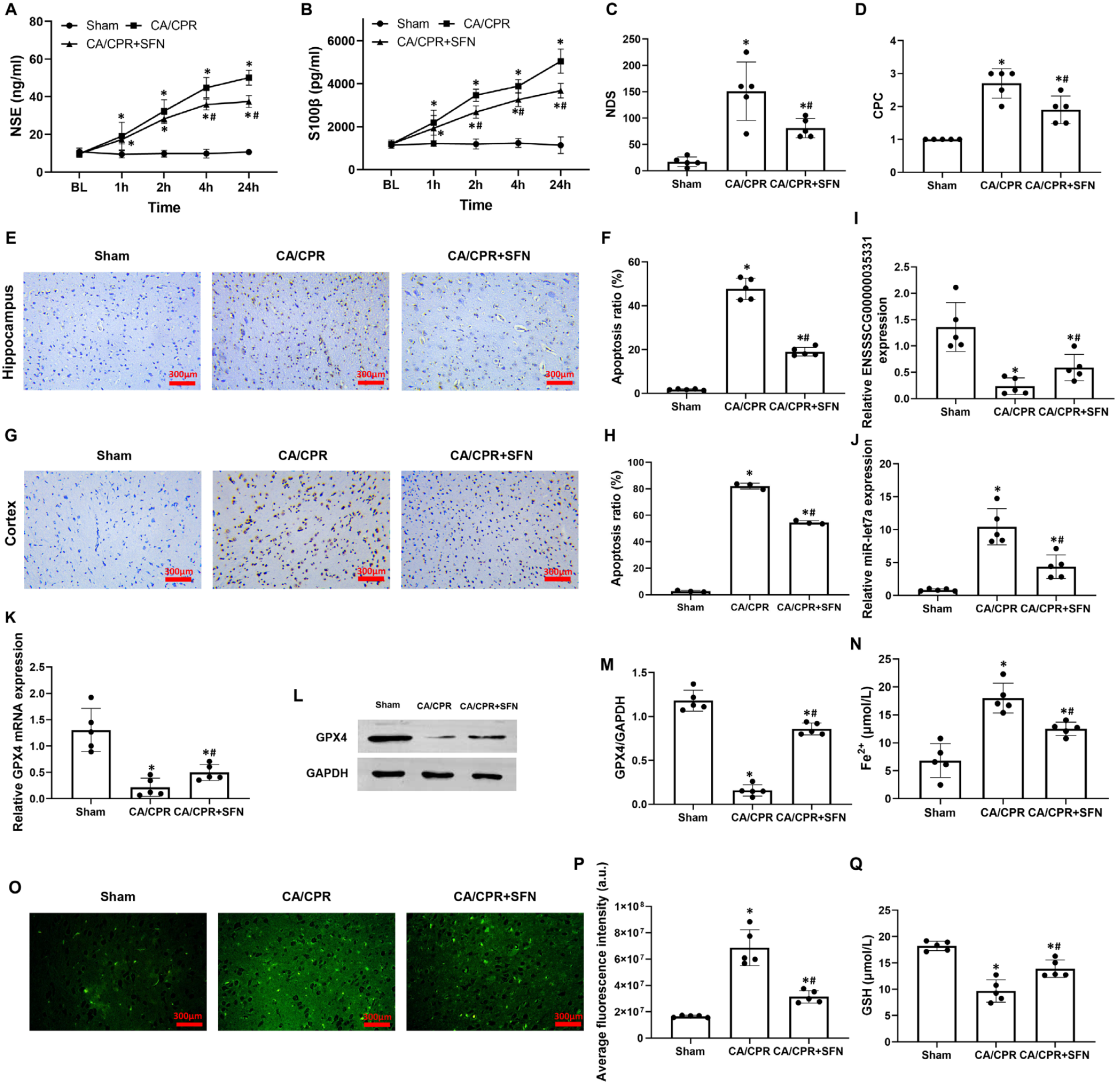
Sulforaphane (SFN) alleviated post‐resuscitation brain injury and hippocampal neuronal ferroptosis possibly by regulating the ENSSSCG00000035331/miR‐let7a/glutathione peroxidase 4 (GPX4) axis. (A, B) Changes of serum concentrations of neuron‐specific enolase (NSE) and S100β protein (S100β) at baseline (BL), and 1 h, 2 h, 4 h, and 24 h post‐resuscitation. (C, D) Evaluation of neurological function using neurological deficit score (NDS) and cerebral performance category (CPC) at 24 h post‐resuscitation. (E–H) Representative photographs of TdT‐mediated dUTP nick‐end labeling staining in hippocampal and cortical tissues at 24 h post‐resuscitation (scale bar = 300 μm, ×200 magnification) and its quantification. (I–K) Relative expression levels of ENSSSCG00000035331 (I), miR‐let7a (J), and GPX4 mRNA (K) in hippocampal tissues at 24 h post‐resuscitation. (L, M) Relative expression levels of GPX4 protein in hippocampal tissues at 24 h post‐resuscitation. (N) Levels of ferrous iron (Fe^2+^) in hippocampal tissues at 24 h post‐resuscitation. (O, P) Representative photographs of immunofluorescence staining of reactive oxygen species (ROS) production in hippocampal tissues at 24 h post‐resuscitation (scale bar = 300 μm, ×200 magnification) and its quantification. (Q) Glutathione (GSH) contents in hippocampal tissues at 24 h post‐resuscitation. CA, cardiac arrest. CPR, cardiopulmonary resuscitation. Each group included 3–5 samples. *p < 0.05 denotes significant differences compared to the Sham group；^#^ *p* < 0.05 denotes significant differences compared to the CA/CPR group.

To further confirm the role of ENSSSCG00000035331 in regulating the miR‐let7a/GPX4 axis‐mediated post‐resuscitation hippocampal neuronal ferroptosis in vivo, ENSSSCG00000035331 was overexpressed in mouse hippocampus using adeno‐associated viruses and then its successful overexpression was confirmed by the IVIS spectrum imaging system (Figure S5). Subsequently, the mouse model of CA and resuscitation was established. Baseline physiological indicators (body weight, heart rate, and body temperature) were not different among the four groups (Figure S6A–C). During CA and resuscitation, those indicators of CPR outcomes including the rates of resuscitation success, duration of CPR, and dosage of epinephrine were uniform in the CA/CPR and CA/CPR + oe‐ENSSSCG00000035331 groups (Figure S6D,E). At 24 h after resuscitation, brain injury biomarkers (NSE, S100β) were significantly increased while neurological functional scores (NFS‐1, NFS‐2) were significantly decreased in the CA/CPR and CA/CPR + oe‐ENSSSCG00000035331 groups when compared with the Sham group. Tissue analysis indicated that pathological injury (tissue disarrangement, cell damage, nucleus pycnosis, and inflammatory infiltration) was significantly severe and cell apoptosis was significantly increased in the hippocampus and cortex in these two groups when compared with the Sham group. However, ENSSSCG00000035331 overexpression significantly alleviated brain injury and neurological dysfunction, and meanwhile significantly reduced hippocampal and cortical pathological injury and apoptosis increased in the CA/CPR + oe‐ENSSSCG00000035331 group when compared to the CA/CPR group (Figure 6A–H, Figure S7A,B). In addition, GPX4 protein expression and GSH contents were significantly decreased while miR‐let7a expression, Fe^2+^ levels, and ROS production were significantly increased in the hippocampus in the CA/CPR and CA/CPR + oe‐ENSSSCG00000035331 groups when compared with the Sham group. Nevertheless, ENSSSCG00000035331 overexpression significantly downregulated miR‐let7a expression, upregulated GPX4 protein expression, and alleviated hippocampal ferroptosis in the CA/CPR + oe‐ENSSSCG00000035331 group when compared to the CA/CPR group (Figure 7I–O). Together with the data from the cell study, we speculated that ENSSSCG00000035331 overexpression alleviated post‐resuscitation brain injury and hippocampal neuronal ferroptosis via regulating the miR‐let7a/GPX4 axis.

### 3.6 Sulforaphane Treatment Alleviated Post‐Resuscitation Brain Injury and Hippocampal Neuronal Ferroptosis Possibly by Regulating the ENSSSCG00000035331/miR‐let7a/GPX4 Axis

Currently, it's difficult to construct porcine hippocampal ENSSSCG00000035331 overexpression and then explore its role in regulating hippocampal ferroptosis via the miR‐let7a/GPX4 axis in a pig model of CA and resuscitation. Considering that the antioxidant, SFN treatment has been confirmed to alleviate cardiac and cerebral IRI by inhibiting GPX4‐mediated ferroptosis [21, 22, 23, 24], the SFN was chosen to investigate its potential regulatory effects on post‐resuscitation hippocampal ferroptosis mediated by the ENSSSCG00000035331/miR‐let7a/GPX4 axis in a pig model.

First, baseline physiological indicators, arterial blood gas, and brain injury biomarkers mentioned above were measured, and no differences in all of them were observed among the Sham, CA/CPR, and CA/CPR + SFN groups (Figure S8A–H, Figure 7A,B). Subsequently, the pig model of CA and resuscitation was similarly established. Consequently, the values of coronary perfusion pressure during CPR and the outcomes of CPR including the duration of CPR, dosage of epinephrine, number of defibrillations, and rate of ROSC were equally obtained, in which no differences were observed between the CA/CPR and CA/CPR + SFN groups (Figure S8I–M). These results indicated that the same baseline and CA/CPR characteristics were achieved in the CA/CPR and CA/CPR + SFN groups.

After resuscitation, the serum levels of NSE and S100β were significantly increased in the CA/CPR and CA/CPR + SFN groups when compared with the Sham group; however, the increases in NSE and S100β were slower in the CA/CPR + SFN group than in the CA/CPR group, in which the serum levels of NSE starting 4 h after resuscitation and the serum levels of S100β starting 2 h after resuscitation were significantly different between the two groups (Figure 7A,B). At 24 h after resuscitation, the scores of NDS and CPC were significantly increased in the CA/CPR and CA/CPR + SFN groups when compared to the Sham group; however, both of them were significantly lower in the CA/CPR + SFN group than in the CA/CPR group (Figure 7C,D). Furthermore, hippocampal and cortical tissue analysis indicated that the ratio of cell apoptosis was significantly higher in the CA/CPR and CA/CPR + SFN groups than in the Sham group; however, SFN treatment significantly decreased both hippocampal and cortical apoptosis when compared to the CA/CPR group (Figure 7E–H). These results indicated that SFN treatment could provide effective post‐resuscitation brain protection in this pig model of CA and resuscitation.

To investigate whether SFN treatment could regulate the ENSSSCG00000035331/miR‐let7a/GPX4 axis and further inhibit post‐resuscitation hippocampal ferroptosis in this pig model, the relative expression levels of ENSSSCG00000035331, miR‐let7a, and GPX4 in hippocampus were measured at 24 h after resuscitation. We observed that ENSSSCG00000035331 expression and GPX4 mRNA and protein expression were significantly decreased while miR‐let7a expression was significantly increased in the hippocampus in the CA/CPR and CA/CPR + SFN groups when compared with the Sham group; however, SFN treatment significantly reversed ENSSSCG00000035331, GPX4, and miR‐let7a expression in hippocampus when compared to the CA/CPR group (Figure 7I–M). Thereafter, those ferroptosis‐related production in the hippocampus was measured at 24 h after resuscitation in pigs. Consequently, Fe^2+^ levels and ROS production were significantly increased while GSH contents were significantly decreased in the hippocampus in the CA/CPR and CA/CPR + SFN groups when compared with the Sham group; however, SFN treatment significantly alleviated post‐resuscitation hippocampal ferroptosis when compared to the CA/CPR group (Figure 7N–Q). These results indicated that SFN treatment could inhibit post‐resuscitation hippocampal ferroptosis possibly through regulating the ENSSSCG00000035331/miR‐let7a/GPX4 axis in pigs.

**FIGURE 8:**
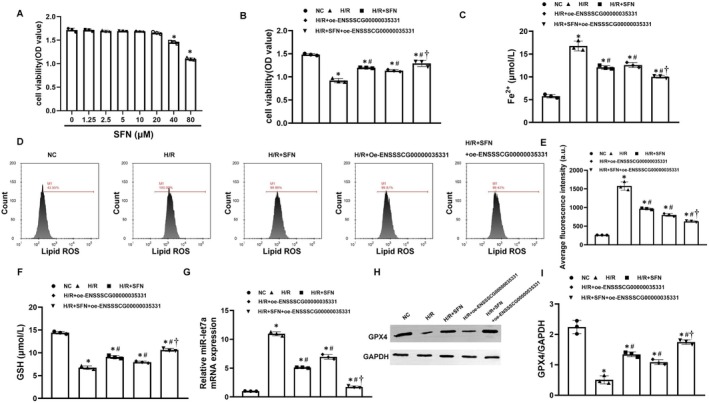
Sulforaphane (SFN) inhibited hypoxia/reoxygenation (H/R)‐induced hippocampal neuronal ferroptosis possibly by regulating the ENSSSCG00000035331/miR‐let7a/glutathione peroxidase 4 (GPX4) axis. (A, B) Cell viability in hippocampal neurons was assessed using CCK‐8 assay. (C) Levels of ferrous iron (Fe^2+^) in hippocampal neurons at 24 h after H/R stimulation. (D, E) Flow cytometric analysis and quantification of lipid reactive oxygen species (ROS) production in hippocampal neurons at 24 h after H/R stimulation. (F) Glutathione (GSH) contents in hippocampal neurons at 24 h after H/R stimulation. (G) Relative expression levels of miR‐let7a in hippocampal neurons at 24 h after H/R stimulation. (H, I) Relative expression levels of GPX4 protein in hippocampal neurons at 24 h after H/R stimulation. NC, normal control. Each group included three replicates. *p < 0.05 denotes significant differences compared to the NC group; #*p* < 0.05 denotes significant differences compared to the H/R group; †*p* < 0.05 denotes significant differences compared to the H/*R* + SFN group or the H/*R* + oe‐ENSSSCG00000035331 group.

To further confirm the role of SFN treatment in regulating the ENSSSCG00000035331/miR‐let7a/GPX4 axis‐mediated hippocampal neuronal ferroptosis, the in vitro study of H/R stimulation was performed in primary porcine hippocampal neurons. Initially, the range of safe concentration of SFN treatment (≤ 20 μM) was confirmed using a cell viability assay (Figure 8A). Subsequently, the concentration of 20 μM of SFN treatment was chosen to use for the remaining experiments. We observed that H/R stimulation significantly downregulated GPX4 protein expression, and decreased GSH contents and cell viability while significantly upregulated miR‐let7a expression, and increased Fe^2+^ levels, and lipid ROS production when compared with the NC group. However, SFN treatment significantly reversed the changes of all those indicators mentioned above in the H/*R* + SFN group when compared to the H/R group. In addition, similar results were observed after H/R stimulation in those hippocampal neurons overexpressing ENSSSCG00000035331. Most importantly, the combination of SFN treatment and ENSSSCG00000035331 overexpression further significantly downregulated miR‐let7a expression, upregulated GPX4 protein expression, and inhibited hippocampal neuronal ferroptosis after H/R stimulation when compared with the H/*R* + SFN group or the H/R + oe‐ENSSSCG00000035331 group (Figure 8B–I). Together with the data from the pig study, we speculated that SFN treatment could alleviate post‐resuscitation brain injury and inhibit hippocampal neuronal ferroptosis by regulating the ENSSSCG00000035331/miR‐let7a/GPX4 axis.

## 4 Discussion

In this study, we utilized a large animal model simulating clinical scenarios to uncover the novel mechanism of ferroptosis regulation in hippocampal neurons after CA and resuscitation. We demonstrated for the first time that a novel lncRNA, ENSSSCG00000035331, significantly protected against post‐resuscitation hippocampal neuronal injury by inhibiting ferroptosis. Mechanistically, ENSSSCG00000035331 exerted its protective effects by antagonizing miR‐let7a, thereby indirectly modulating the expression of key genes such as GPX4. In addition, treatment with the antioxidant SFN resulted in significantly higher ENSSSCG00000035331 and GPX4 expression, markedly lower miR‐let7a expression, and further alleviated post‐resuscitation hippocampal neuronal ferroptosis and brain injury. These results indicated that those therapeutic approaches by targeting lncRNAs might provide effective post‐resuscitation brain protection in the clinical setting of CA and resuscitation.

Recent research on CA and resuscitation has shown that the overall prognosis remains grim [25, 26]. Post‐resuscitation brain injury is recognized as one of the key factors contributing to the high morbidity and mortality [27]. Regrettably, effective treatment options for such neurological injuries remain scarce. Ferroptosis, a novel form of programmed cell death driven by iron‐dependent lipid peroxidation reactions, has gained significant attention in recent years across various disease models, particularly in the realm of research on brain IRI [25, 26, 27]. Of particular interest is the exceptional sensitivity of certain specific neuronal populations (e.g., CA1 neurons in the hippocampus, cortical, cerebellar, striatal, and thalamic neurons) to IRI, likely closely associated with their heightened susceptibility to ferroptosis [28, 29]. In the present study, we successfully established a pig model of CA and resuscitation using electrical defibrillation, meticulously monitoring various baseline physiological parameters throughout the experiment to ensure the precision and credibility of the model construction. Despite all experimental pigs regaining spontaneous circulation, those animals in the CA/CPR group exhibited more severe neurological dysfunction (NDS and CPC), suggesting potential adverse effects of the resuscitation process on porcine cognition and other neural functions. In addition, laboratory test data indicated that when compared to the Sham group, the pigs in the CA/CPR group have significantly elevated serum levels of brain injury biomarkers (NSE and S100β), concurrent with neuronal reduction and exacerbated apoptosis in hippocampal neurons. Collectively, these findings confirmed substantive damage to neurons during the resuscitation process. Further investigation revealed distinct features of ferroptosis in damaged hippocampal tissues after resuscitation, characterized by increased Fe^2+^ levels, heightened oxidative stress (manifested by elevated ROS production), decreased GSH contents, and downregulated expression of ferroptosis‐suppressing genes. The evidence suggested that ferroptosis may be involved in the damage process of these neurons within hours to days following CA and resuscitation.

LncRNA is a novel type of RNA molecule defined as being longer than 200 nucleotides and has been shown to effectively regulate gene expression at both the transcriptional and translational levels [28]. Recent evidence suggests that lncRNAs are associated with neurological dysfunction following CA and resuscitation. Notably, lncRNA GAS5 inhibits miR‐137, promoting INPP4 expression, thereby suppressing PI3K/Akt pathway activation and leading to cell apoptosis and inflammation, which are involved in the hypoxic response axis of astrocyte‐microglia crosstalk following CA and resuscitation [29]. Additionally, lncRNA‐PS is a critical driver of ShcA activation, leading to cognitive impairment in mice following CA and resuscitation [30]. Despite the identification of an increasing number of lncRNAs through transcriptome sequencing analysis, which may be involved in the pathological process of post‐resuscitation brain injury [31], unfortunately, to date, the functions of most lncRNAs and their specific regulatory mechanisms remain unclear. In our study, following bioinformatics analysis of hippocampal tissues from both the CA/CPR and Sham groups, our focus shifted to a differentially expressed lncRNA, designated ENSSSCG00000035331. Given these premises, we postulated that ENSSSCG00000035331 served as a pivotal regulator in the progression of brain injury following CA and resuscitation, specifically in the context of ferroptosis mediation. Neuronal loss resulting from ferroptosis is closely linked to a variety of neurological dysfunctions, such as memory decline and cognitive deficits. Moreover, it may impede self‐repair following brain injury by disrupting neural regeneration processes, such as inhibiting proliferation, differentiation, and migration of neural stem cells. Interestingly, we found that ENSSSCG00000035331 overexpression significantly enhanced the survival capability of porcine hippocampal neurons after H/R stimulation, concurrently reducing intracellular ROS production and further reducing the incidence of cell ferroptosis.

Previous scientific investigations have convincingly demonstrated that the functionality and mechanisms of lncRNAs are intimately tied to their precise subcellular localization [20]. Particularly in the context of IRI, interactions with miRNAs in the cytoplasm have emerged as a central regulatory axis. This perspective is grounded in a wealth of experimental evidence illustrating how cytoplasmic lncRNAs can act as “molecular sponges,” sequestering miRNAs and relieving their suppressive effects on target mRNAs, thereby influencing post‐transcriptional gene expression regulation [32]. Given the specific enrichment of ENSSSCG00000035331 in the cytoplasm of neuronal cells as revealed by RNA‐FISH, we were led to hypothesize that this lncRNA potentially exerted its influence at the cytoplasmic level by directly engaging in or modulating miRNA‐mediated post‐transcriptional regulatory networks, thereby impacting the expression of target genes.

Using bioinformatics tools, including miRanda and RNAhybrid software for Venn diagram analysis, we successfully identified miR‐let7a as a potential downstream target of ENSSSCG00000035331. Previous studies have shown that elevated miR‐let7a expression levels could inhibit cell proliferation and increase intracellular ROS production [33]. Furthermore, miR‐let7a downregulates key proteins involved in glucose metabolism and changes closely associated with the ferroptosis process, implying that miR‐let7a promotes ferroptosis by modulating redox homeostasis and energy metabolism pathways [34]. Going further, miR‐let7a can directly or indirectly regulate the expression of key genes in the ferroptosis process, such as targeting glutamine transporter SLC1A5 to modulate ferroptosis in melanoma cells [35]. As SLC1A5 is a transporter mediating glutamine uptake, its function is inhibited by miR‐let7a, leading to disrupted glutamine metabolism, exacerbating iron‐dependent lipid peroxide accumulation and the occurrence of ferroptosis [36]. Through dual luciferase reporter assays, we confirmed miR‐let7a as a key downstream target of ENSSSCG00000035331 and uncovered an interaction between the two molecules. Our experimental data showed that the suppression of miR‐let7a expression facilitated to alleviate hippocampal neuronal ferroptosis after H/R stimulation.

To further investigate how miR‐let7a mediates the regulation of post‐resuscitation hippocampal neuronal ferroptosis by ENSSSCG00000035331, we examined the downstream target genes of the typical miRNA pathway. We employed miRanda and RNAhybrid software to deeply mine the miRNA‐mRNA interaction network, revealing a potential regulatory relationship between miR‐let7a and GPX4 mRNA. GPX4 is an antioxidant enzyme that reduces phospholipid hydroperoxides in membranes, thereby protecting cells against ferroptosis [37]. In our study, we further enriched this theory by demonstrating that miR‐let7a could directly act on GPX4 mRNA, suppressing its gene expression, and thus aggravating neuronal ferroptosis induced by H/R stimulation. These findings collectively revealed the central position of ENSSSCG00000035331 in the ferroptosis regulatory network and suggested that the therapeutic strategies targeting ENSSSCG00000035331 might have a positive impact on mitigating post‐resuscitation brain injury.

Currently, nuclear factor erythroid 2‐related factor 2 (Nrf2), known as a classic and key antioxidant regulatory gene, has been confirmed to be involved in the regulation of ferroptosis in various diseases [38]. Especially, the Nrf2/GPX4 axis has been proven to play an important role in inhibiting neuronal ferroptosis after regional cerebral IRI [39, 40]. In addition, the SFN used as a Nrf2 activator has been shown to promote GPX4 expression to inhibit the ferroptosis, and finally ameliorate cerebral injury after ischemic stroke [23]. Thus, SFN treatment could be a potential therapeutic medication for alleviating IRI‐induced neuronal ferroptosis via regulating the Nrf2/GPX4 axis, but its regulatory mechanism remains to be investigated. In the present study, the newly discovered lncRNA ENSSSCG00000035331 was shown to recover GPX4 expression by interacting with miR‐let7a, and finally inhibit neuronal ferroptosis after CA and resuscitation. Additionally, the inhibition of miR‐let7a expression was shown to be an effective neuroprotective approach after regional cerebral IRI in several studies [41, 42]. Based on the evidence above, we attempted to investigate the regulatory effect of SFN treatment on the ENSSSCG00000035331/miR‐let7a/GPX4 axis‐mediated hippocampal neuronal ferroptosis after CA and resuscitation. In our in vivo study, a dose of 2 mg/kg of SFN treatment was chosen to administer at 5 min after resuscitation, which has been shown to exert effective protective effects by inhibiting cardiac ferroptosis and lung pyroptosis after CA and resuscitation in pigs [22, 43]. In our in vitro study, a feasible concentration of 20 μM of SFN treatment was obtained using a cell viability assay. Our results showed that SFN treatment significantly increased ENSSSCG00000035331 and GPX4 expression while decreased miR‐let7a expression and neuronal ferroptosis in in vitro and in vivo studies. Hence, SFN treatment could inhibit post‐resuscitation hippocampal neuronal ferroptosis by regulating the ENSSSCG00000035331/miR‐let7a/GPX4 axis.

Our study had several limitations. First, we focused on tissue sampling and analysis at specific time points after resuscitation, but did not systematically track the dynamic evolution of ferroptosis and its regulatory mechanisms across different time stages following CA and resuscitation. Second, our observation set a short‐term to intermediate‐term post‐resuscitation period so that the evaluation of long‐term (weeks to months) aspects was lacked, such as neurological function recovery, scar formation, and neural regeneration. Third, although our study revealed the critical role of ENSSSCG00000035331 in ferroptosis regulation through its interactions with miR‐let7a and GPX4 in a series of cell and mouse experiments, but our mechanistic investigation was difficult to implement in a pig model. Fourth, the use of pigs as experimental animals led to high costs and a limited sample size. Fifth, although we explored the effect of SFN treatment on the signaling axis mentioned above, it remains unclear whether this axis plays a dominant role.

## 5 Conclusions

Our study demonstrated that a novel lncRNA, ENSSSCG00000035331, could alleviate post‐resuscitation brain injury and hippocampal neuronal ferroptosis by regulating the miR‐let7a/GPX4 axis. In addition, the SFN, as a potent antioxidant, could produce effective post‐resuscitation brain protection possibly by inhibiting neuronal ferroptosis via regulating the ENSSSCG00000035331/miR‐let7a/GPX4 axis.

## Author Contributions

Xingui Wu and Jiefeng Xu designed the study. Mao Zhang, Wenbin Zhang, Ziwei Chen, Lu He, Qijiang Chen, Pin Lan, Lulu Li, and Xianlong Wu performed the experiments and recorded the data. Mao Zhang and Wenbin Zhang analyzed the data and wrote the manuscript.

## Conflicts of Interest

The authors declare no conflicts of interest.

## Supporting information


**Figure S1.** Baseline characteristics and cardiopulmonary resuscitation (CPR) outcomes in the first pig study. (A–H) Baseline body weight, heart rate, mean arterial pressure, end‐tidal CO_2_, pH, PCO_2_, PO_2_, and lactate. (I–M) Coronary perfusion pressure, duration of CPR, dosage of epinephrine, number of defibrillations, and animal number of ROSC. CA, cardiac arrest. ROSC, return of spontaneous circulation. Each group included three samples.

**Figure S2.** Verification of five top differentially expressed lncRNAs in hippocampal tissues in the pig study. (A–E) Relative expression levels of ENSSSCG00000031079, ENSSSCG00000040228, ENSSSCG00000032037, ENSSSCG00000034990, and ENSSSCG00000038924 in hippocampal tissues at 24 h post‐resuscitation. CA, cardiac arrest. CPR, cardiopulmonary resuscitation. Each group included three samples.

**Figure S3.** Confirmation of ENSSSCG00000035331 overexpression in primary porcine hippocampal neurons. (A) Representative photographs of primary porcine hippocampal neuron culture. (B, C) Confirmation of ENSSSCG00000035331 transfection with adeno‐associated viruses by fluorescence microscopy (scale bar = 300 μm, ×200 magnification).

**Figure S4.** LncRNA ENSSSCG00000035331 overexpression alleviated hippocampal neuronal damage after hypoxia/reoxygenation (H/R) stimulation. (A, B) Cell viability and lactate dehydrogenase (LDH) levels in hippocampal neurons at 24 h after H/R stimulation. (C–F) Flow cytometric analysis and quantification of cell apoptosis and cytosolic reactive oxygen species (ROS) production in hippocampal neurons at 24 h after H/R stimulation. NC, normal control. Each group included three replicates. *p < 0.05 denotes significant differences compared to the NC group; #*p* < 0.05 denotes significant differences compared to the H/R group.

**Figure S5.** Confirmation of ENSSSCG00000035331 overexpression in mouse hippocampus. ENSSSCG00000035331 overexpression was constructed in mouse hippocampus using mCherry‐tagged adeno‐associated viruses and then confirmed by IVIS spectrum imaging system. Each group included six samples.

**Figure S6.** Baseline characteristics and cardiopulmonary resuscitation (CPR) outcomes in the mouse study. (A–C) Baseline body weight, heart rate, and body temperature. (D, E) Duration of CPR and dosage of epinephrine. CA, cardiac arrest. Each group included six samples.

**Figure S7.** Evaluation of pathological injury of hippocampal and cortical tissues in the mouse study. (A, B) Representative photographs of hematoxylin and eosin staining in hippocampal and cortical tissues at 24 h post‐resuscitation (Scale bar = 300 μm, ×200 magnification). CA, cardiac arrest; CPR, cardiopulmonary resuscitation. Each group included three samples.

**Figure S8.** Baseline characteristics and cardiopulmonary resuscitation (CPR) outcomes in the second pig study. (A–H) Baseline body weight, heart rate, mean arterial pressure, end‐tidal CO_2_, pH, PCO_2_, PO_2_, and lactate. (I–M) Coronary perfusion pressure, duration of CPR, dosage of epinephrine, number of defibrillations, and animal number of ROSC. SFN, sulforaphane; CA, cardiac arrest; ROSC, return of spontaneous circulation. Each group included five samples.

**Table S1.** Primer sequences.

**Table S2.** The biotin‐labeled probe sequences used in this study.

## Data Availability

The data that support the findings of this study are available from the corresponding author upon reasonable request.
