# Supplementary figures and images for: LncRNA ENSSSCG00000035331 Alleviates Hippocampal Neuronal Ferroptosis and Brain Injury Following Porcine Cardiopulmonary Resuscitation by Regulating the miR‐let7a/GPX4 Axis
Source: CNS Neurosci Ther. 2025 Apr 16;31(4):e70377. doi: 10.1111/cns.70377 (PMC12001066; doi:10.1111/cns.70377)

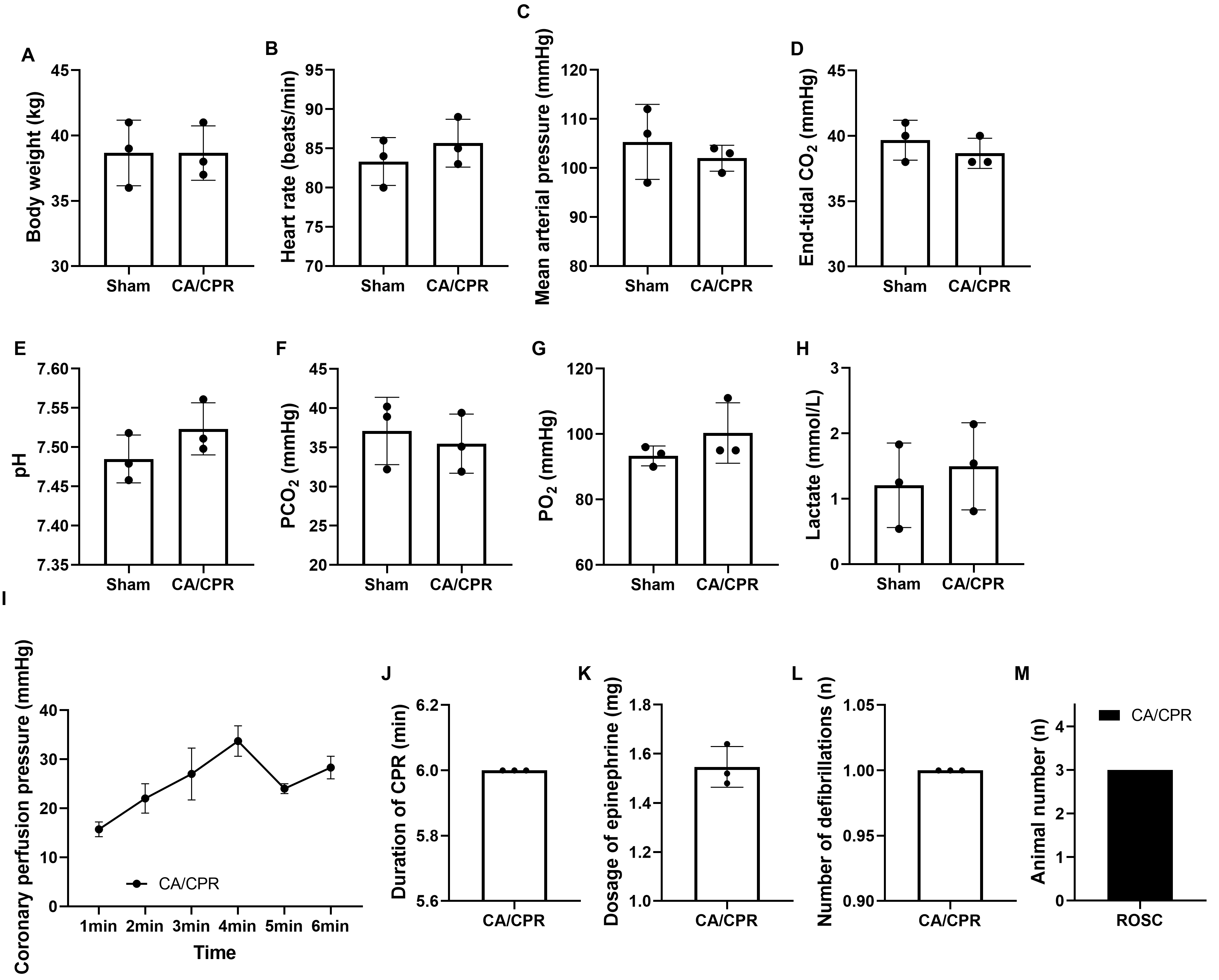

Supplement: Supplementary file 1 — Figure S1. Baseline characteristics and cardiopulmonary resuscitation (CPR) outcomes in the first pig study. (A–H) Baseline body weight, heart rate, mean arterial pressure, end‐tidal CO2, pH, PCO2, PO2, and lactate. (I–M) Coronary perfusion pressure, duration of CPR, dosage of epinephrine, number of defibrillations, and animal number of ROSC. CA, cardiac arrest. ROSC, return of spontaneous circulation. Each group included three samples. [file CNS-31-e70377-s003.tif]

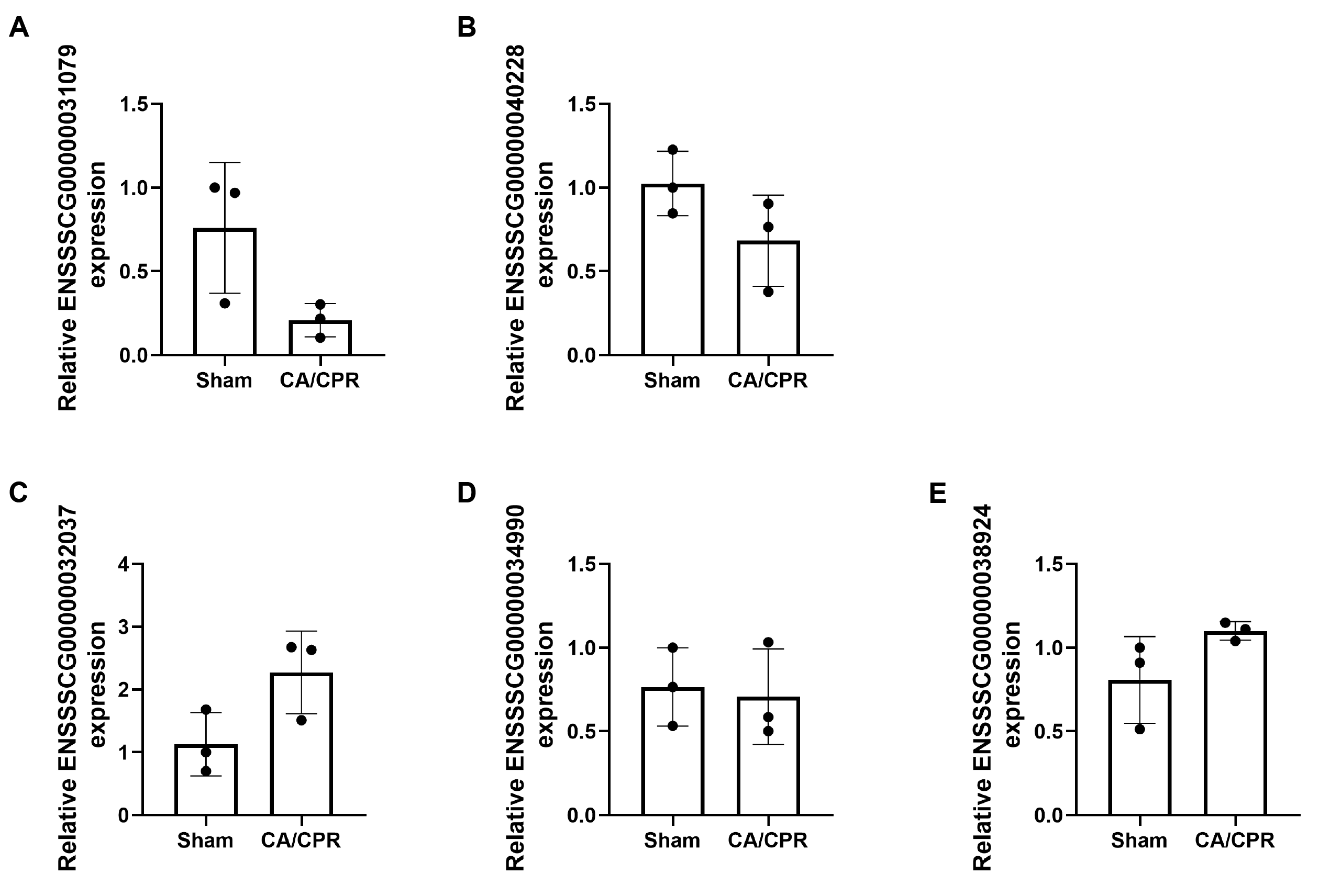

Supplement: Supplementary file 2 — Figure S2. Verification of five top differentially expressed lncRNAs in hippocampal tissues in the pig study. (A–E) Relative expression levels of ENSSSCG00000031079, ENSSSCG00000040228, ENSSSCG00000032037, ENSSSCG00000034990, and ENSSSCG00000038924 in hippocampal tissues at 24 h post‐resuscitation. CA, cardiac arrest. CPR, cardiopulmonary resuscitation. Each group included three samples. [file CNS-31-e70377-s004.tif]

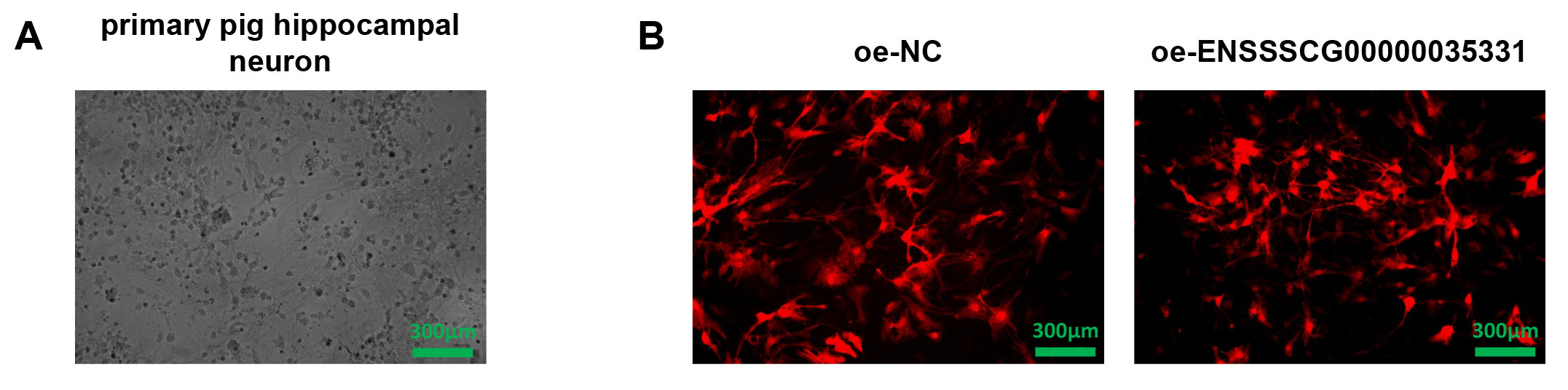

Supplement: Supplementary file 3 — Figure S3. Confirmation of ENSSSCG00000035331 overexpression in primary porcine hippocampal neurons. (A) Representative photographs of primary porcine hippocampal neuron culture. (B, C) Confirmation of ENSSSCG00000035331 transfection with adeno‐associated viruses by fluorescence microscopy (scale bar = 300 μm, ×200 magnification). [file CNS-31-e70377-s005.tif]

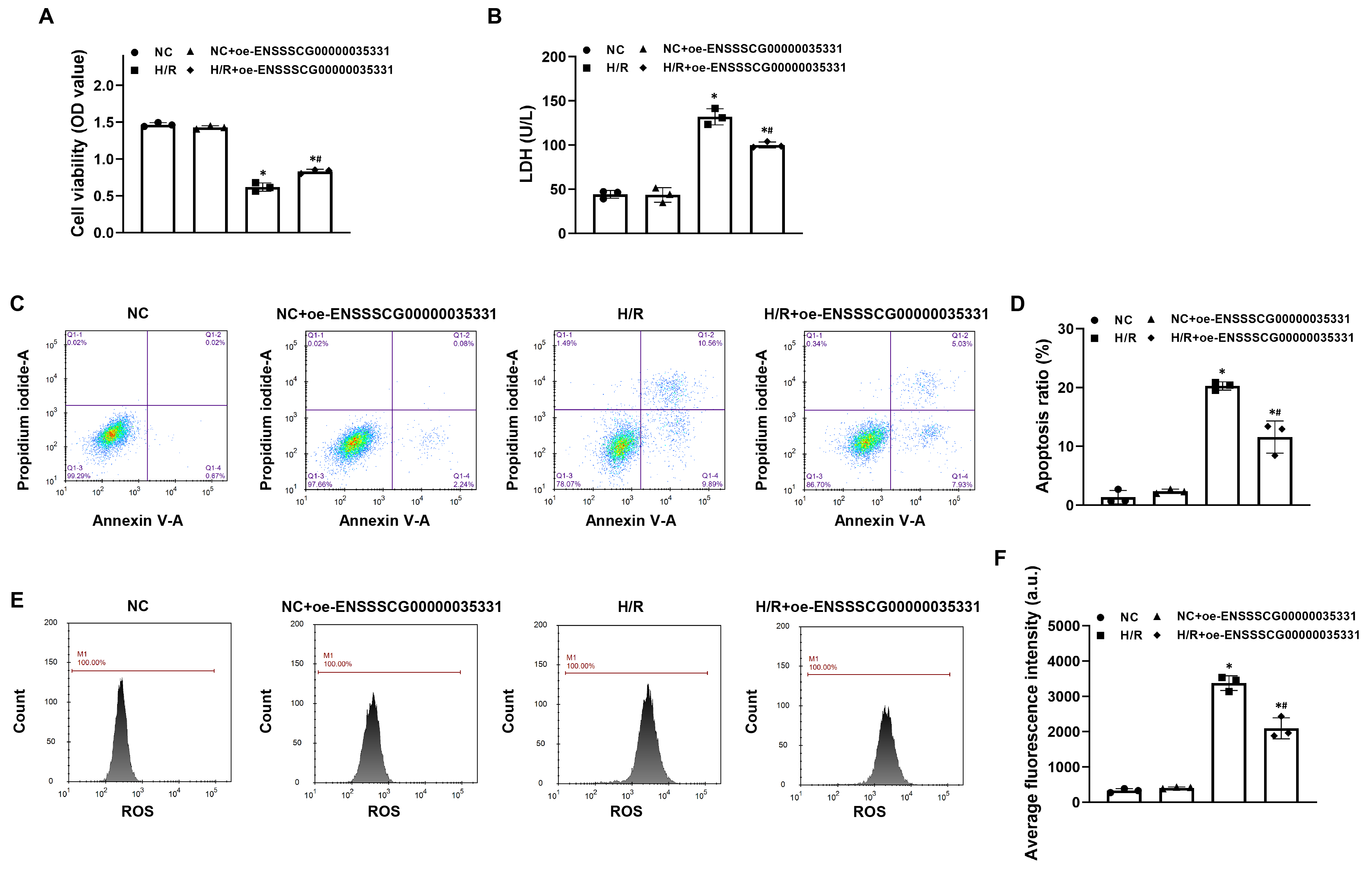

Supplement: Supplementary file 4 — Figure S4. LncRNA ENSSSCG00000035331 overexpression alleviated hippocampal neuronal damage after hypoxia/reoxygenation (H/R) stimulation. (A, B) Cell viability and lactate dehydrogenase (LDH) levels in hippocampal neurons at 24 h after H/R stimulation. (C–F) Flow cytometric analysis and quantification of cell apoptosis and cytosolic reactive oxygen species (ROS) production in hippocampal neurons at 24 h after H/R stimulation. NC, normal control. Each group included three replicates. *p < 0.05 denotes significant differences compared to the NC group; #p < 0.05 denotes significant differences compared to the H/R group. [file CNS-31-e70377-s002.tif]

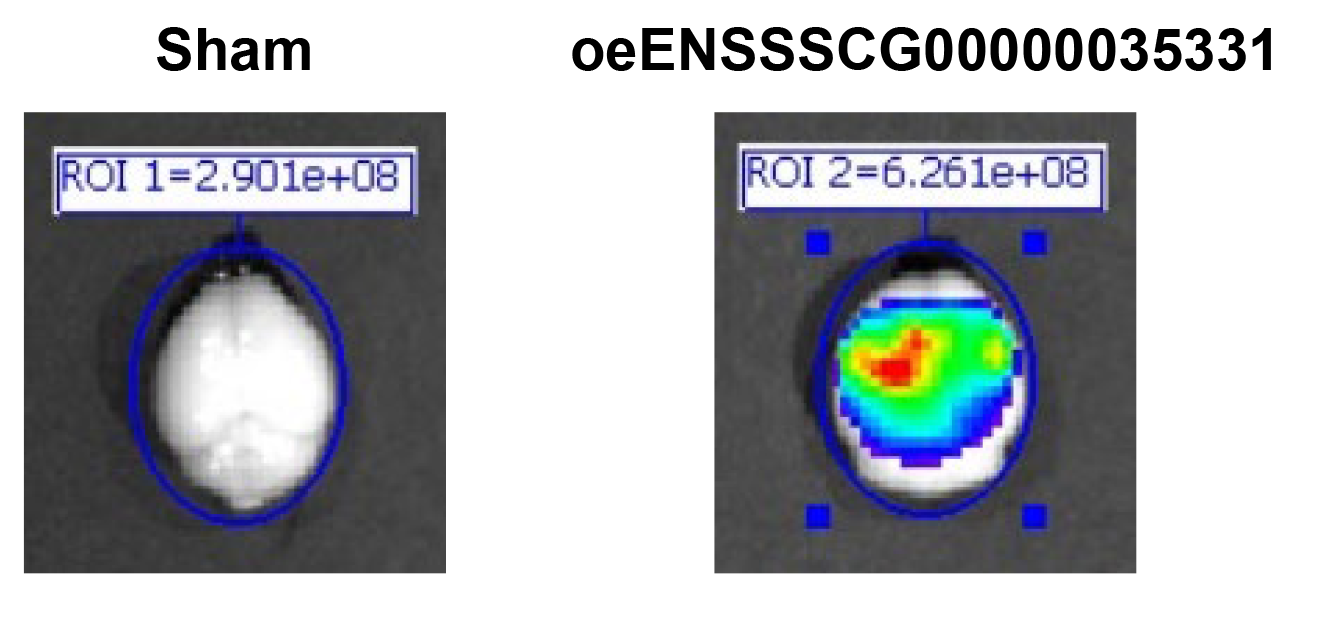

Supplement: Supplementary file 5 — Figure S5. Confirmation of ENSSSCG00000035331 overexpression in mouse hippocampus. ENSSSCG00000035331 overexpression was constructed in mouse hippocampus using mCherry‐tagged adeno‐associated viruses and then confirmed by IVIS spectrum imaging system. Each group included six samples. [file CNS-31-e70377-s007.tif]

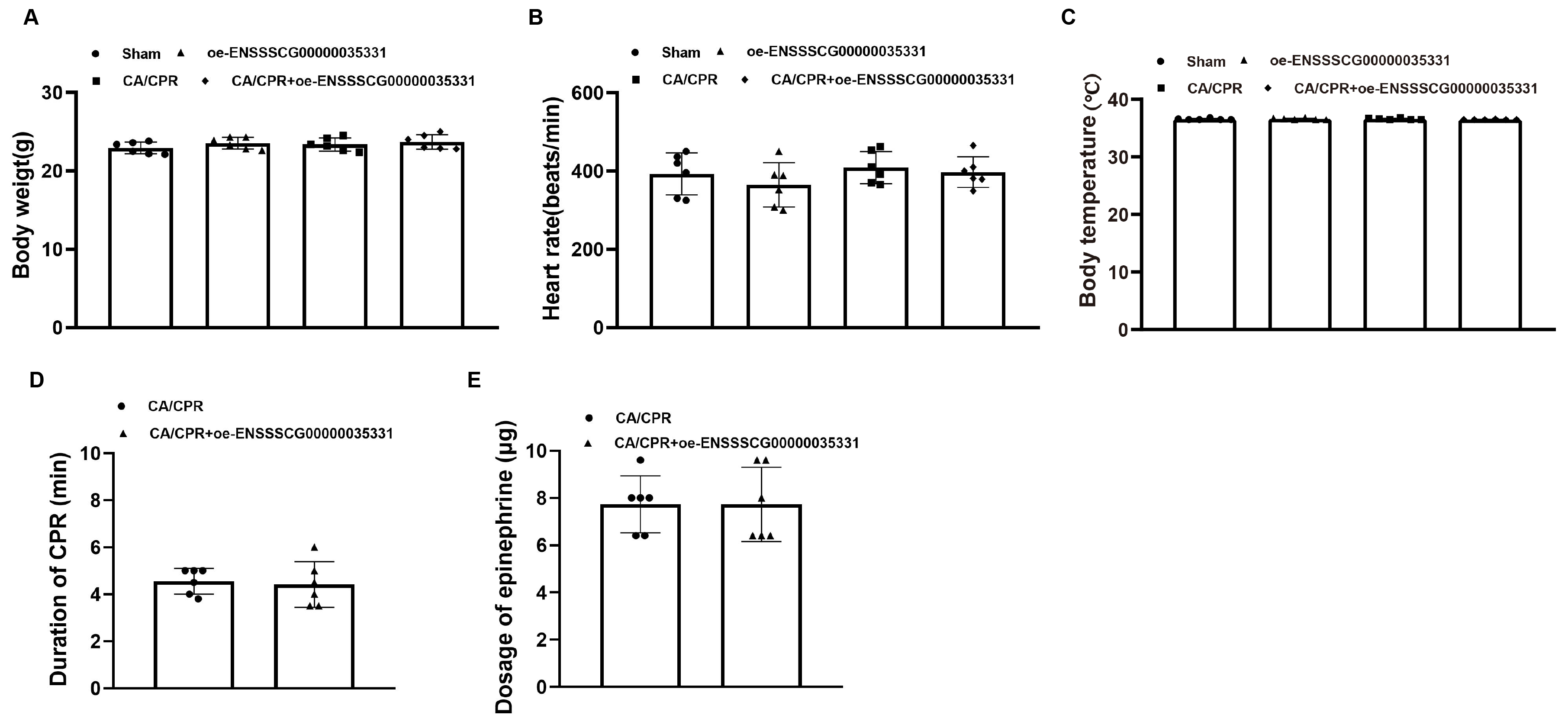

Supplement: Supplementary file 6 — Figure S6. Baseline characteristics and cardiopulmonary resuscitation (CPR) outcomes in the mouse study. (A–C) Baseline body weight, heart rate, and body temperature. (D, E) Duration of CPR and dosage of epinephrine. CA, cardiac arrest. Each group included six samples. [file CNS-31-e70377-s008.tif]

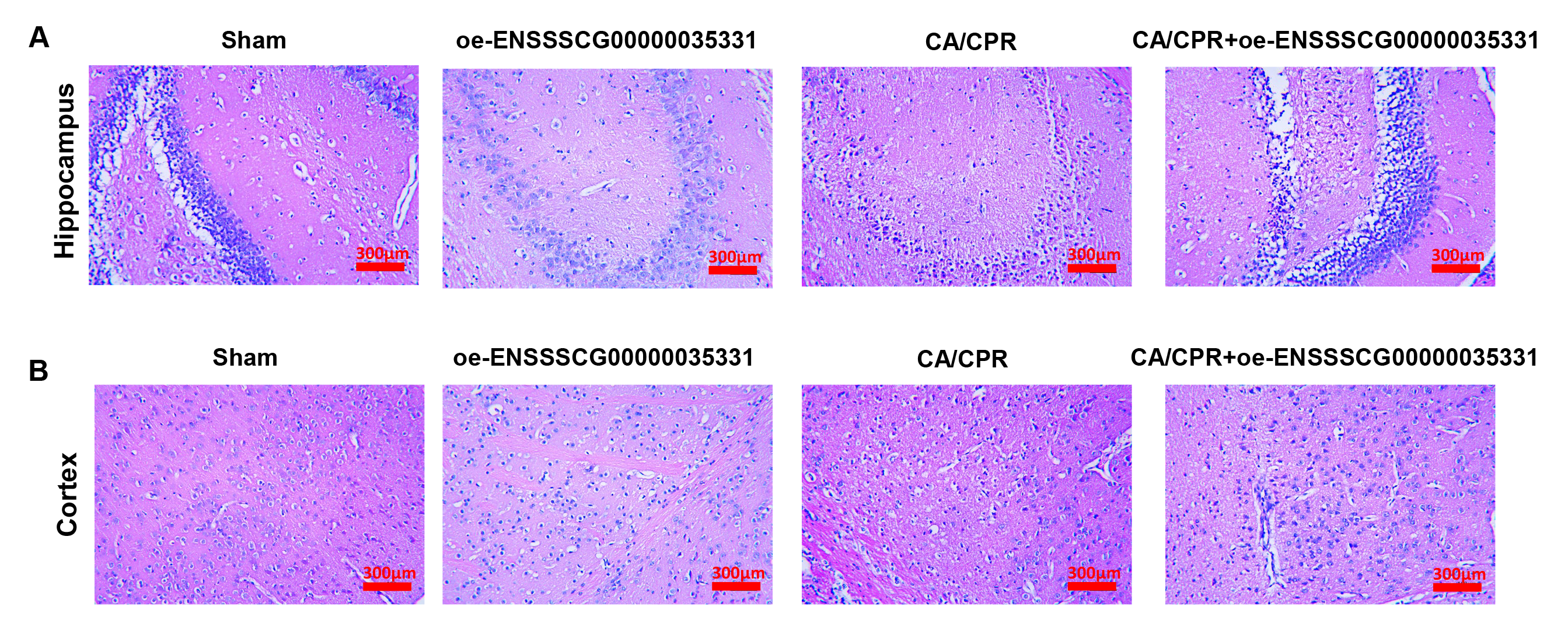

Supplement: Supplementary file 7 — Figure S7. Evaluation of pathological injury of hippocampal and cortical tissues in the mouse study. (A, B) Representative photographs of hematoxylin and eosin staining in hippocampal and cortical tissues at 24 h post‐resuscitation (Scale bar = 300 μm, ×200 magnification). CA, cardiac arrest; CPR, cardiopulmonary resuscitation. Each group included three samples. [file CNS-31-e70377-s009.tif]

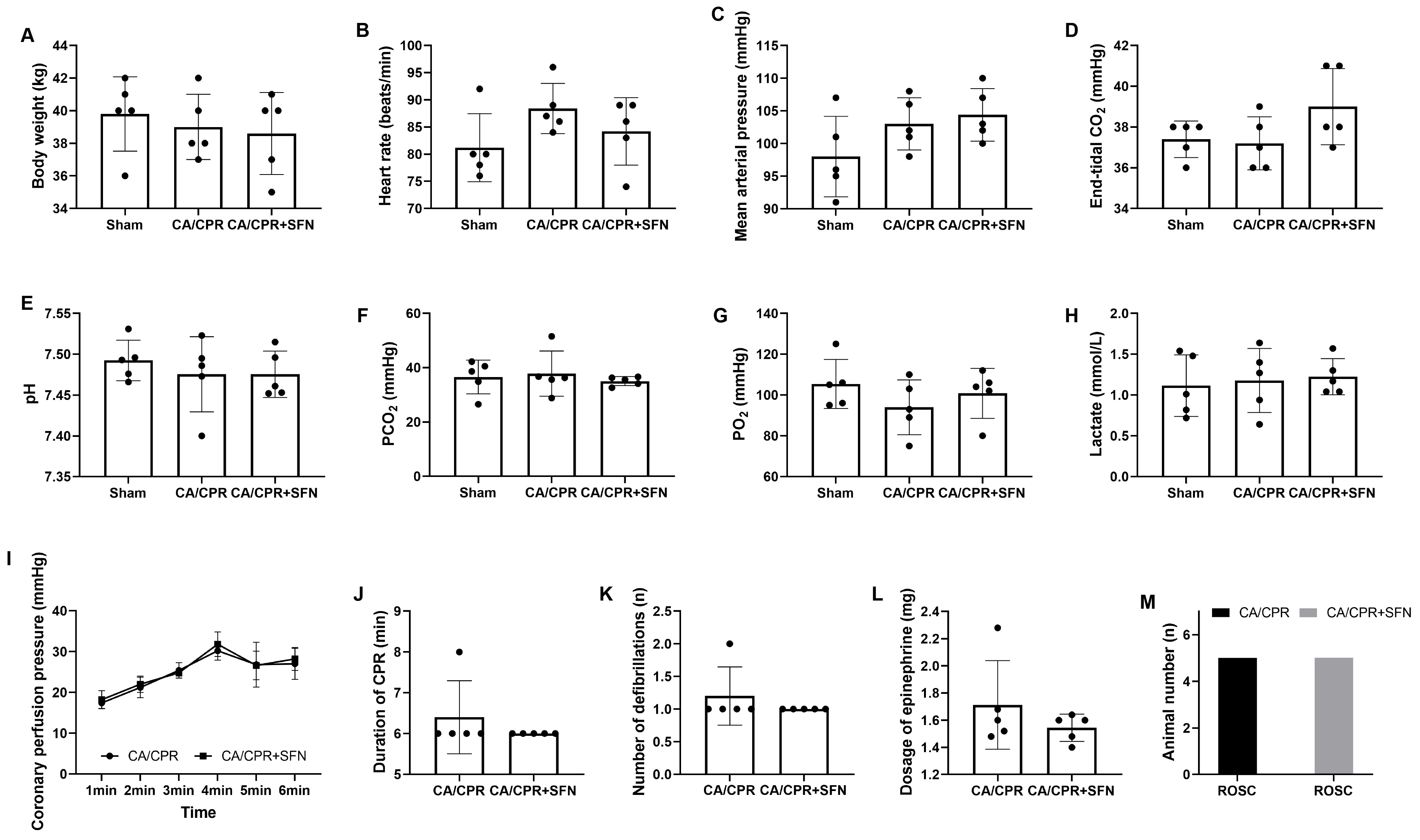

Supplement: Supplementary file 8 — Figure S8. Baseline characteristics and cardiopulmonary resuscitation (CPR) outcomes in the second pig study. (A–H) Baseline body weight, heart rate, mean arterial pressure, end‐tidal CO2, pH, PCO2, PO2, and lactate. (I–M) Coronary perfusion pressure, duration of CPR, dosage of epinephrine, number of defibrillations, and animal number of ROSC. SFN, sulforaphane; CA, cardiac arrest; ROSC, return of spontaneous circulation. Each group included five samples. [file CNS-31-e70377-s010.tif]
