# Supplementary material for: LncRNA ENSSSCG00000035331 Alleviates Hippocampal Neuronal Ferroptosis and Brain Injury Following Porcine Cardiopulmonary Resuscitation by Regulating the miR‐let7a/GPX4 Axis
Source: CNS Neurosci Ther. 2025 Apr 16;31(4):e70377. doi: 10.1111/cns.70377 (PMC12001066; doi:10.1111/cns.70377)
Supplement: Supplementary file 9 — Table S1. Primer sequences. [file CNS-31-e70377-s001.docx]

**Table 1. Primer Sequences**

| primers |  | forward | reverse |
| --- | --- | --- | --- |
| GPX4 | pig | TGGCCTCTCAATGAGGCAAG | CCCTTGGGCTGGACTTTCAT |
| FTH1 | pig | GAATTTGCGCTGCACGTGGT | AGAGATACTCGGCCATGCCA |
| ACSL4 | pig | AGATCTGTTCGCTGTTGCTGA | AGAGCCCGCCACACAAGTTA |
| NOX1 | pig | TGTTGCTGGTCATGCGGTAT | AGCATTTACGCAGGCTCCTT |
| COX2 | pig | AGACAGCATAAACTGCGCCT | CATCATCAGACCAGGCACCA |
| GAPDH | pig | TCGGAGTGAACGGATTTGGC | TGACAAGCTTCCCGTTCTCC |
| ENSSSCG00000032037 | pig | CCACTGGAGAGCTTTCAGCA | ACTCATTGTGGGAGCAGACG |
| ENSSSCG00000034990 | pig | TAACTGCCCTGCCAAGAGTG | CTTCCTCAACCTTCCTGCGT |
| ENSSSCG00000038924 | pig | CTCGGGACCAGGTGATCAAAA | CCTGAGACCCCTTCCTTTGG |
| ENSSSCG00000031079 | pig | CTGGAAGAGCTAAGCCACCC | GGCCATGTTGGTACTGGGAA |
| ENSSSCG00000035331 | pig | TGTCTTGCTTCCGGTCACTC | CTTGTGGTCGCTTCCTGACT |
| ENSSSCG00000040228 | pig | AGCAGATGATAGGCGGATGC | GCTGCTTTTTGCAGGAGGTC |
| U6 | Pig | CTCGCTTCGGCAGCACA | AACGCTTCACGAATTTGCGT |
| ssc-let7a | Pig | GCGCTGAGGTAGTAGGTTGT | TGCAGGGTCCGAGGTAT |
| let7a Reverse transcription | Pig | GTCGTATCCAGTGCAGGGTCCGAGGTATT  CGCACTGGATACGAAACTAT | |
