# Supplementary material for: LncRNA ENSSSCG00000035331 Alleviates Hippocampal Neuronal Ferroptosis and Brain Injury Following Porcine Cardiopulmonary Resuscitation by Regulating the miR‐let7a/GPX4 Axis
Source: CNS Neurosci Ther. 2025 Apr 16;31(4):e70377. doi: 10.1111/cns.70377 (PMC12001066; doi:10.1111/cns.70377)
Supplement: Supplementary file 10 — Table S2. The biotin‐labeled probe sequences used in this study. [file CNS-31-e70377-s006.docx]

**Table 2. The biotin-labeled probe sequences used in this study**

|  | Sequence 5’-3’ |
| --- | --- |
| NC-1 | CCTGGCTTTCTTATGGACGC-biotin |
| NC-2 | GACCTCCGCGTAGTTTGTTC-biotin |
| NC-3 | TGTCGGTAATGCCCGTCCTT-biotin |
| NC-4 | TCCCGTCTTGTGCTTGAACG-biotin |
| NC-5 | TCTACAGGCTCTTCGTTGGC-biotin |
| Lnc ENSSSCG00000035331-1 | GGTCTCCCAGTGTTTGCACT-biotin |
| Lnc ENSSSCG00000035331-2 | CTTGGGAGCCACTTGGGATT-biotin |
| Lnc ENSSSCG00000035331-3 | CGGTGTCCTTTCTGTGACCA-biotin |
| Lnc ENSSSCG00000035331-4 | TCCCCAAGAGCCAAACACTC-biotin |
| Lnc ENSSSCG00000035331-5 | CAAGACTTCCGTTCCCCTCC-biotin |
| GPX4-1 | CCTTGGCTGAGAATTCGTGC-biotin |
| GPX4-2 | TTGGTGACGATGCACACGTA-biotin |
| GPX4-3 | TGCAAGGGAAGGCCAGAATC-biotin |
| GPX4-4 | AGGCTCAGCACACACTTGTT-biotin |
| GPX4-5 | GGGGCTGGTTTTTAGGCAGA-biotin |
